# Karyoptosis mediates cell death and neurodegeneration upon proteotoxic stress

**DOI:** 10.1038/s41467-026-73802-w

**Published:** 2026-06-25

**Authors:** Rebecca Casterton, Aitana Martinez-Cotrina, Jodi Barnard, Eleanor Wycherley, Yanling Hu, Rhys Anderson, Sebastien Janel, Jiin Byun, Olivia Houghton, Daniel A. Solomon, Juan Alcalde, Frank Lafont, Marc-David Ruepp, Frank Hirth, Bart Tummers, Yong-Yeon Cho, Gian De Nicola, Sarah Mizielinska, Manolis Fanto

**Affiliations:** 1 Department of Basic and Clinical Neuroscience, King’s College London, London, UK; 2 UK Dementia Research Institute at King’s College London, London, UK; 3 Guy’s & St. Thomas’ Hospital NHS Foundation Trust, London, UK; 4 Univ. Lille, CNRS, Inserm, CHU Lille, Institut Pasteur Lille, U1019 - UMR 9017 - CIIL - Center for Infection and Immunity of Lille, Lille, France; 5 College of Pharmacy, The Catholic University of Korea, Seoul, Republic of Korea; 6 Centre for Inflammation Biology and Cancer Immunology, King’s College London, London, UK; 7 Randall Division of Cell and Molecular Biophysics, King’s College London, London, UK

**Keywords:** Cell death in the nervous system, Macroautophagy, Neurodegeneration

## Abstract

Neurodegenerative diseases are frequently associated with proteotoxic stress linked to disease specific proteins. The autophagy-lysosome system provides essential control of proteotoxic stress and its failure can lead to initiation of apoptosis. However, in aging and neurodegenerative diseases apoptosis is insufficient to account for all neuronal death, and several different cell death types have been reported in these contexts. Here we show that karyoptosis, a distinct form of cell death, can be induced by proteotoxic stress and then develops through nuclear degeneration and cellular expulsion of nuclear material. We establish that karyoptosis is regulated by the p38 kinase signalling pathway, which controls stability of the nuclear lamina protein LaminB1 via direct phosphorylation. We demonstrate that karyoptosis affects neurons in models of amyotrophic lateral sclerosis/frontotemporal dementia (ALS/FTD) pathology. Finally, we identify karyoptotic features in *post-mortem* frontal cortex of FTD and Alzheimer’s disease (AD) patients. Together these findings characterise a form of cell death directly linked to proteotoxic stress and nuclear lamina stability that is associated with neurodegeneration.

## Abstract (web summary)

This study characterizes karyoptosis as a mechanism of neuronal cell death triggered by proteotoxic stress and executed through disintegration of the nuclear lamina, which may account for the death of a significant proportion of neurons in dementia.

## Introduction

Neurons are highly specialised for long-term post-mitotic survival and are therefore particularly vulnerable to proteotoxic stress, relying heavily on pathways for the maintenance of proteostasis. The autophagy-lysosome system is essential for maintaining cellular and protein homeostasis and its impairment has several important links with disease and cell death^1,2^. Understanding the specific mechanisms that link these processes is crucial to inform the development of effective treatment strategies to prevent neuronal loss in neurological disorders^1,2^.

The significance of the autophagy-lysosome system is particularly notable in neurodegenerative disease pathogenesis. A key hallmark of all major age-related neurodegenerative diseases is that they are closely associated with proteotoxic stress due to the accumulation and toxic aggregation of disease-specific proteins^3^. Indeed, genes encoding components of the system’s machinery are frequently associated with diseases of the nervous system^4^ and autophagy-lysosome efficiency is known to decrease with age^5^. Combined, these factors result in an ineffective autophagy-lysosome system that is overwhelmed by the misfolded protein aggregates^6^, which can ultimately underpin neuronal cell death in disease.

As a pro-survival pathway, when the autophagy-lysosome system is impaired, it can trigger the intracellular switch to programmed cell death through initiation of apoptotic pathways, and similarly, apoptosis activation often coincides with a coordinated shutdown of autophagy^7–11^. However, post-mitotic neurons are adapted to be resistant to apoptosis^12^. In the context of neurodegenerative disease, this resistance generates difficulty in identification of a clear, single cell death mechanism responsible for neuronal cell death in disease. Indeed, cell death in the absence of apoptosis signalling has been detected in different models and diseases^13–15^, including confirmation of specific non-apoptotic mechanisms such as ferroptosis^16^ and necroptosis^17^. It is likely that the exact mechanisms responsible for neuronal cell death in neurodegenerative disease varies, both between and within different diseases, according to vulnerable neuronal sub-populations and intracellular pathological burdens. Understanding the precise contribution of each cell death mechanism in neurodegenerative disease is crucial, as it enables preservation of neuronal populations affected in disease with targeted precision.

In this work, we set out to determine the mechanism of neuronal cell death stemming from proteotoxic stress associated with impairment of autophagic lysosomal clearance. In doing so, we identified a distinct type of non-apoptotic and non-necrotic cell death, which we name ‘karyoptosis’. Our results demonstrate that karyoptosis is triggered by proteotoxic stress, and first manifests through destabilization of the nuclear lamina, which leads to nuclear degeneration, cell atrophy and ultimately death. We identify phosphorylation of the nuclear lamina protein, LaminB1, by the p38 mitogen activated kinases (MAPK) as a key regulatory step mediating nuclear lamina degeneration and subsequent cell survival in karyoptosis. Finally, we detect features of karyoptosis in post-mortem frontal cortex of individuals with dementia, through unbiased single-cell clustering analysis of LaminB1 properties and morphological features. Furthermore, this method enables us to quantitatively estimate a significant effect size of karyoptosis in neurodegenerative disease.

## Results

### Inhibition of autophagic lysosomal clearance causes nuclear degeneration and expulsion of nuclear material in large extracellular vesicles

**Fig. 1:**
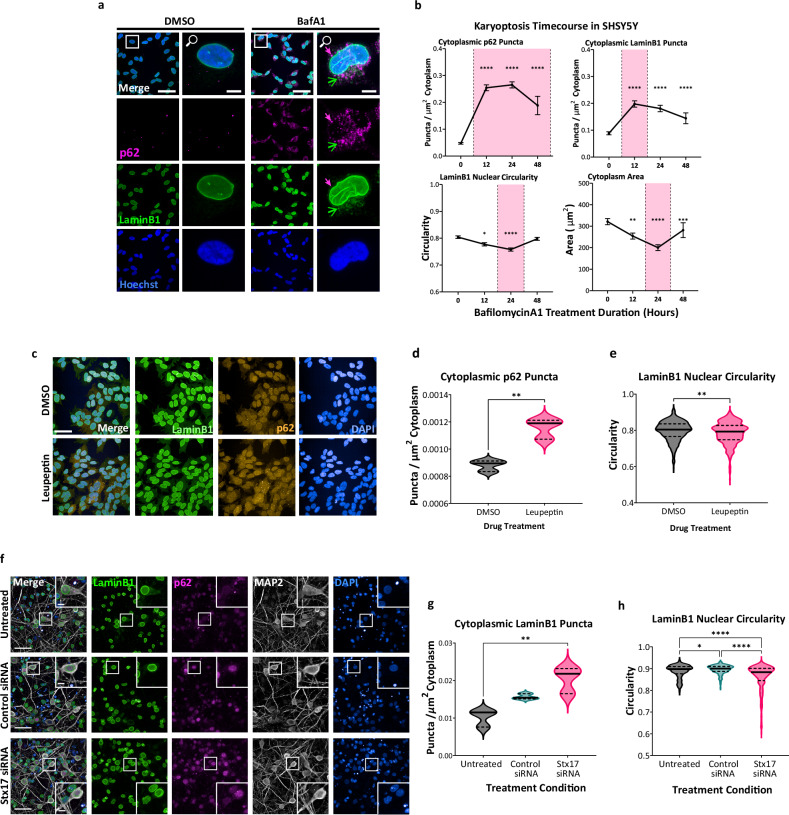
Impairment of autophagic clearance triggers loss of nuclear degeneration and expulsion and cell death. **a** Images of p62, LaminB1 and Hoechst immunofluorescence in SH-SY5Y cells treated with DMSO or 10 nM BafilomycinA1 for 24 h. White dotted box = zoom. **b** Quantification of cytoplasmic p62 puncta, cytoplasmic LaminB1 puncta, LaminB1 nuclear circularity and cytoplasm area in cells treated with BafilomycinA1 for increasing time durations. Pink shading highlights peaks and troughs for each karyoptosis variable. **c** Images of p62, LaminB1 and Hoechst immunofluorescence in SH-SY5Y cells treated with Leupeptin. Quantification of cytoplasmic p62 puncta (**d**), and LaminB1 nuclear circularity (**e**). **f** Images of p62, LaminB1, DAPI and MAP2 immunofluorescence in rat primary cortical neurons treated with either control or Syntaxin17 siRNA for 72 h. **g** Quantification of cytoplasmic LaminB1 puncta and **h** LaminB1 circularity. Scale bars: 50 µm (**a**, **c**, **f**) or 10 µm (**a** and **c**: inset). Data presented as mean ± SEM (**b**, **g**, **k**, **m**), and in violin plot solid lines represent median, dashed lines interquartile range (**d**, **e**, **g**, **h**). Statistics: Kruskal–Wallis test and Dunn’s post-hoc multiple comparisons test (**b**, **h**); unpaired *t*-test (**d**), Mann–Whitney test (**e**), one-way ANOVA and Tukey’s multiple comparisons test (**g**);* *p* < 0.05, ** *p* < 0.01, *** *p* < 0.001, ****p < 0.0001. *N* (cells)—**b**: 0 h = 118, 12 h = 82, 24 h = 86, 48 h = 87); **e** DMSO = 474, Leupeptin = 409; **h** untreated = 276, control siRNA = 279, Stx17 siRNA = 278; (wells) = **d**, **g** 3 per condition. Kruskal–Wallis statistic = 216.0, *p* < 0.0001 & Dunn’s multiple comparisons: 0 vs. 12 *p* < 0.0001, 0 vs. 24 *p* < 0.0001, 0 vs. 48 *p* < 0.0001 (**b**, p62 puncta); Kruskal–Wallis statistic = 77.63, *p* < 0.0001 & Dunn’s multiple comparisons: 0 vs. 12 *p* < 0.0001, 0 vs. 24 *p* < 0.0001, 0 vs. 48 *p* < 0.0001 (**b**, Lamin B1 puncta); Kruskal–Wallis statistic = 42.15, *p* < 0.0001 & Dunn’s multiple comparisons: 0 vs. 12 *p* = 0.0143, 0 vs. 24 *p* < 0.0001, 0 vs. 48 *p* > 0.9999 (**b**, Lamin B1 circularity); Kruskal–Wallis statistic = 45.62, *p* < 0.0001 & Dunn’s multiple comparisons: 0 vs. 12 *p* = 0.0089, 0 vs. 24 *p* < 0.0001, 0 vs. 48 *p* = 0.0002 (**b**, Cytoplasm area); *t* = 5.661, *p* = 0.0048 (**d**); *U* = 107987, *p* = 0.0045 (**e**); *F* = 13.02, *p* = 0.0066 & Tukey’s multiple comparisons: untreated vs. control siRNA *p* = 0.0793, Untreated vs Stx17 siRNA *p* = 0.0053, control siRNA vs. Stx17 siRNA *p* = 0.1158 (**g**); Kruskal–Wallis statistic = 69.52, *p* < 0.0001 & Dunn’s multiple comparisons: untreated vs. control siRNA *p* = 0.0251, untreated vs. Stx17 siRNA *p* < 0.0001, control siRNA vs. Stx17 siRNA *p* < 0.0001 (**h**). Raw data and further statistical information provided in the Source Data File and at 10.5281/zenodo.19220863.

Proteotoxic stress often leads to generalised loss of cellular homeostasis and cell death. To study the mechanistic details of this process, we have first exploited chronic pharmacological impairment of lysosomal clearance of autophagosomes and other cellular components, as an efficient method of proteotoxic stress induction. As we have previously shown^18^, a chronic low-dose treatment of BafilomycinA1 (BafA1), an inhibitor of autophagic lysosomal clearance, induces the same response in multiple cell types, whereby puncta of the nuclear lamina protein, LaminB1, accumulate in the cytoplasm and a regular nuclear shape is lost (Fig. 1a and Supplementary Fig. 1a–c). These features are accompanied by cell shrinkage, the cytoplasmic presence of nuclear membrane components such as nuclear pore proteins, and increased elasticity of both the cytoplasm and nuclear surface, measured by atomic force microscopy (Supplementary Fig. 1a, d–g). Analysis of these phenomena in neuroblastoma cells over time suggests that cytoplasmic LaminB1 puncta accumulation is transient and peaks at 12 h post-stressor in this model, before nuclear lamina shape abnormalities and cytoplasm atrophy occur (Fig. 1b). These features co-occur with cytoplasmic accumulation of the autophagy receptor, p62, in puncta partially distinct from those of LaminB1. This accumulation of p62 demonstrates impaired autophagic clearance and widespread proteotoxic stress (Fig. 1a, b). The same phenomena are observed across multiple models of autophagic clearance impairment, including neuroblastoma cells treated with the lysosomal protease inhibitor Leupeptin (Leu) (Fig. 1c–e) and in rat primary cortical neurons by knock down of *STX17*, encoding for Syntaxin-17, an essential component for autophagic clearance^19^ (Fig. 1f–h).

**Fig. 2:**
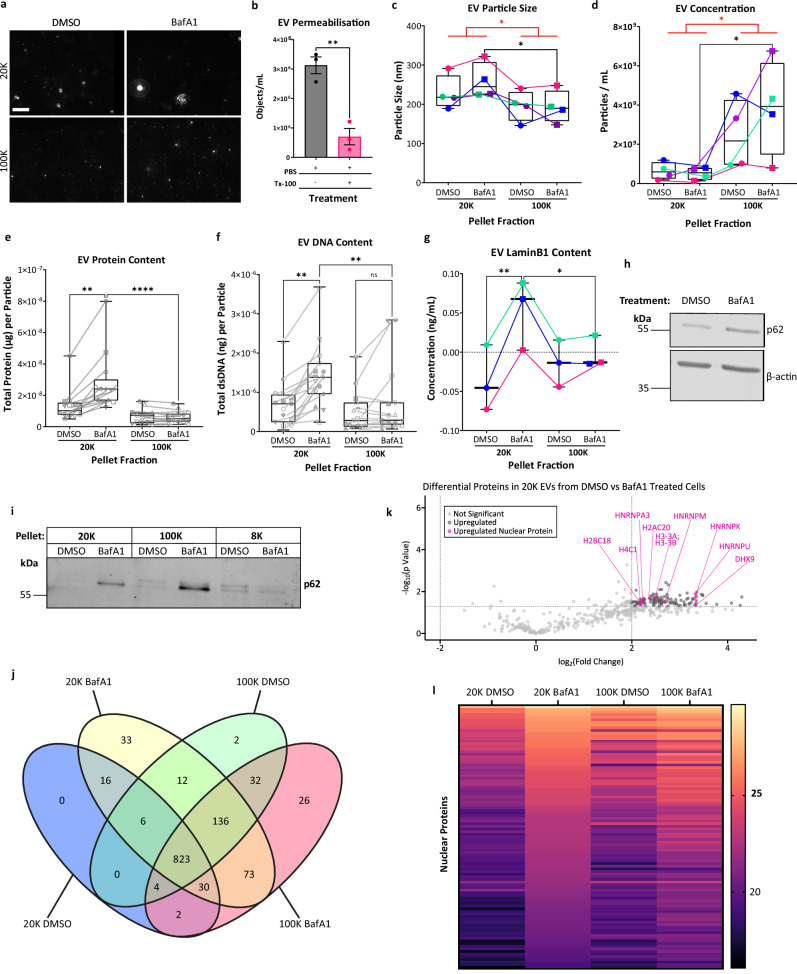
Karyoptotic EV properties and content. **a** Representative video stills of extracellular vesicles (EV) obtained from 20 K and 100 K EV fractions isolated from the media of SH-SY5Y treated with 0.1% DMSO or 20 nM BafA1 for 24 h. Scale bar: 1 µm (**a**). **b** NTA particle quantification following Triton ×-100 permeabilization of EVs. EV size (**c**) and concentration (**d**) from SH-SY5Y treated with DMSO or BafA1 for 24 h. Total protein (**e**) and double-stranded DNA (**f**) content in EV fraction samples, normalised to particle count. **g** ELISA quantification of LaminB1 concentration in EV fractions isolated from media SH-SY5Y cells treated with DMSO or BafA1. **h** Western blot for p62 and β-actin in lysate of SH-SY5Y cells and **i** in EV media fractions (“8 K”: cell debris and apoptotic bodies). **j** Venn diagram overview of unique and shared proteomics hits between different EV sample types. **k** Volcano plot comparing proteomics analysis of 20 K EV fractions. Dark grey = significantly upregulated proteins, pink = significantly upregulated nuclear proteins. Dotted lines indicate thresholds for statistical significance (*p* < 0.05) and fold-change ( ± 2). **l** Heat map of significantly changing nuclear proteins determined from GO analysis of proteomics data (higher abundance = lighter, lower abundance = darker). Data presented as mean ± SEM (**b**). Box plots denote median and interquartile range, whiskers minimum and maximum values (**c–g**). Statistics: two-tailed unpaired *t*-test (**b**, **k**). two-way ANOVA and Tukey’s post-hoc (**c–g**), two-tailed Welch *t*-test for the 20 K vs 100 K overall comparison (red, **c**, **d**). * *p* < 0.05, ** *p* < 0.01, **** *p* < 0.0001. *N* (independent experiments per condition)—**b** = 3; **c**, **d** = 4; **e** = 13; **f** = 15; **g** = 3; coloured lines connect samples from the same experiment, highlighting the trends (**c–g**); **k** = 3. *t* = 6.110, *p* = 0.0036 (**b**); row factor (conditions) *F* (3, 9) = 11.58, *p* = 0.0019, column factor (replicates) *F* (3, 9) = 7.701, *p* = 0.0074 & Tukey’s multiple comparisons: BafA1 20 K vs. BafA1 100 K *p* = 0.0110; (red) *t* = 2.396, *p* = 0.0316 (**c**); row factor (conditions) *F* (3, 9) = 2.403, *p* = 0.1350, column factor (replicates) *F* (3, 9) = 5.793, *p* = 0.0174 & Tukey’s multiple comparisons: BafA1 20 K vs. BafA1 100 K *p* = 0.0258; (red) *t* = 3.397, *p* = 0.0106 (**d**); row factor (conditions) *F* (12, 36) = 2.852, *p* = 0.0074, column factor (replicates) *F* (3, 36) = 16.41, *p* < 0.0001& Tukey’s multiple comparisons: DMSO 20 K vs. BafA1 20 K *p* = 0.0011, BafA1 20 K vs. BafA1 100 K *p* < 0.0001 (**e**); row factor (conditions) *F* (14, 42) = 3.856, *p* = 0.0003, column factor (replicates) *F* (3, 42) = 8.110, *p* = 0.0002 & Tukey’s multiple comparisons: DMSO 20 K vs. BafA1 20 K *p* = 0.0043, BafA1 20 K vs. BafA1 100 K *p* = 0.0063, DMSO 100 K vs. BafA1 100 K *p* = 0.6724 (**f**); row factor (conditions) *F* (2, 6) = 16.94, *p* = 0.0034, column factor (replicates) *F* (3, 6) = 17.04, *p* = 0.0024 & Tukey’s multiple comparisons: DMSO 20 K vs. BafA1 20 K *p* = 0.0019, BafA1 20 K vs. BafA1 100 K *p* = 0.0215 (**g**); see Supplementary Data 1 for data in (**k**). Raw data and further statistical information provided in the Source Data File and at 10.5281/zenodo.19221077.

LaminB1 has an extremely long half-life and autophagic clearance is the only protein degradation pathway currently known to affect LaminB1 levels^20,21^. As autophagic clearance was inhibited in our models, but LaminB1 levels were reduced (see below), this led us to speculate that LaminB1 and other nuclear components could be re-routed for clearance via cellular expulsion, similarly to what has been reported in other models of neurodegeneration and cancer^22–24^. In neuroblastoma cells treated with BafA1 for 24 h, release of Triton-sensitive extracellular vesicles (EVs) (Fig. 2a, b) is detected concurrently with the intracellular changes previously observed in this model. We therefore decided to characterise these EVs to determine if they could be a viable extracellular expulsion route for LaminB1.

We utilised sequential ultra-centrifugation to isolate a fraction (centrifuged at 20,000 × *g* denoted as “20 K“) enriched in larger EVs, and a separate fraction (centrifuged at 100,000 × *g*, “100 K”) containing smaller exosome-type EVs from media of cells treated with either BafA1 or DMSO (Fig. 2a–c). While representing a minority of the released EVs (Fig. 2d), the 20 K EV fraction obtained from BafA1-treated cells is uniquely richer in DNA and proteins (Fig. 2e, f) and includes more LaminB1 and p62 (Fig. 2g–i). A full proteomics study (Supplementary Data 1 and Fig. 2j) of these EVs confirms enrichment in histones, hnRNPs and other nuclear proteins in the 20 K EV fraction obtained from cells where autophagic clearance has been impaired by BafA1 treatment (Fig. 2k, l). Additionally, 20 K EVs obtained from BafA1 treated cells contain proteins which are below detection threshold from EVs released from DMSO treated cells. This includes LaminB1, but also other DNA damage, transcription and DNA topology proteins like MSH6, SAFB, and TOP2B (Supplementary Data 1). These findings affirm that when lysosomal autophagic clearance is chronically impaired, the observed intracellular changes to LaminB1 are accompanied by a dynamic outflow of nuclear material and protein stressors from the cell via EVs.

### Nuclear degeneration is caused by proteotoxic stress and constitutes an independent mechanism of cell death termed karyoptosis

**Fig. 3:**
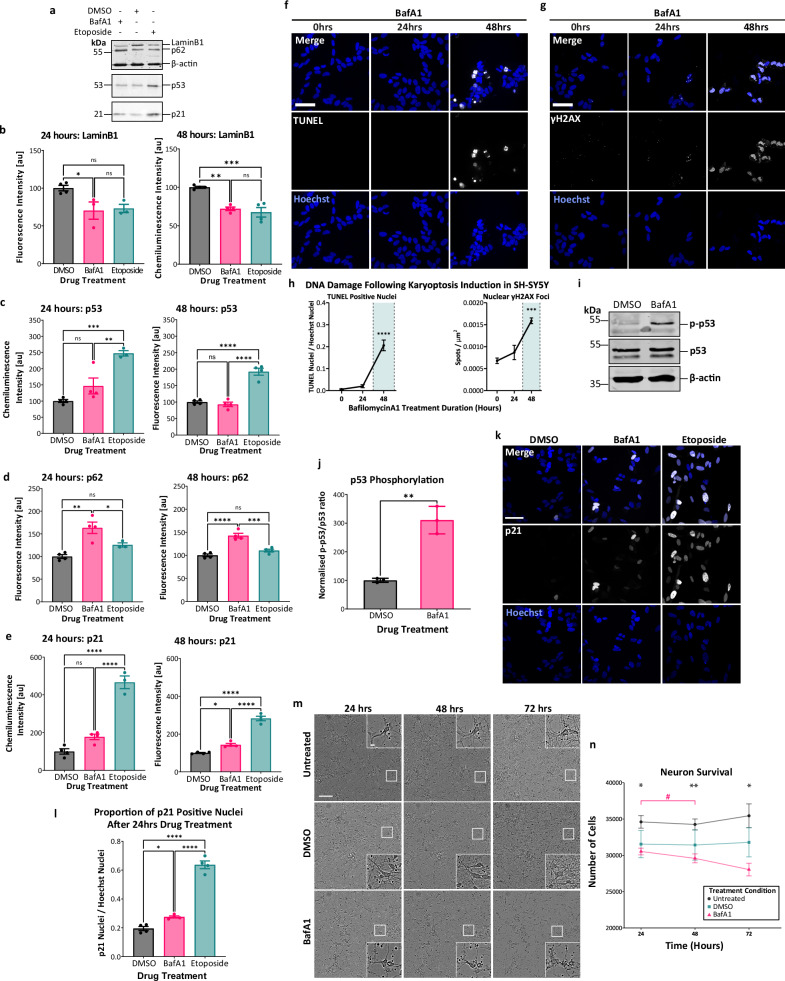
Karyoptosis shares some features with but is distinct from DNA damage-induced senescence. **a** Western blot for LaminB1, p62, β-actin (loading control), p53 and p21 from cell lysate of SH-SY5Y cells treated with BafilomycinA1 or etoposide for 24 h. Quantification of protein levels for LaminB1 (**b**), p53 (**c**), p62 (**d**), and p21 (**e**) at 24 or 48 h. **f** Images of immunofluorescence for DNA damage associated with cell death (TUNEL assay) and **g** the DNA damage marker γH2AX, in SH-SY5Y cells treated with BafA1 for increasing time duration. **h** Proportion of TUNEL-positive nuclei and quantification of nuclear γH2AX foci in SH-SY5Y cells treated with BafilomycinA1. Green shading highlights peaks for each. **i** Western blot for p53 and phospho-p53 (p-p53) from whole cell lysate of SH-SY5Y treated with BafA1. **j** Quantification of p-p53:p53 ratio. **k** Immunofluorescence images of p21 in SH-SY5Y cells treated with BafilomycinA1 or etoposide. **l** Quantification of proportion of p21-positive nuclei. **m** Live brightfield images of rat primary cortical neurons captured every 24 h following DMSO or BafilomycinA1 treatment. **n** Quantification of neuronal cell count of rat primary cortical neurons from live imaging. Scale bars: 50 µm (**f**, **g**, **k**, **m**), 10 µm (**m**, inset). Data presented as mean *± *SEM (**b–e**, **h**, **j**, **l**, **n**). Statistics: two-tailed unpaired *t*-test (**j**); one-way ANOVA and Tukey’s post-hoc (**b–e**, **l**); one-way ANOVA and Dunnett’s post-hoc (**h**); two-way ANOVA and Tukey’s post-hoc (**n**) * *p* < 0.05, ** *p* < 0.01, *** *p* < 0.001, **** *p* < 0.0001. *N* (independent experiments per condition): **b–e** = 4 except (**b**) 24 h BafA1 and etoposide, **c–e** 24 h etoposide, which = 3; **j** = 3; *N* (wells): **h** = 4 per time point except 3 for γH2AX 0 and 24 h; **l**, **m** = 4 per condition. *F* = 6.019, *p* = 0.0301 & Tukey’s multiple comparisons: DMSO vs. BafA1 *p* = 0.0422, DMSO vs. Etoposide *p* = 0.063, BafA1 vs. Etoposide *p* = 0.9594 (**b**, 24 h); *F* = 20.35, *p* = 0.0005 & Tukey’s multiple comparisons: DMSO vs. BafA1 *p* = 0.0017, DMSO vs. Etoposide *p* = 0.0006, BafA1 vs. Etoposide *p* = 0.6984 (**b**, 48 h); *F* = 19.26, *p* = 0.0009 & Tukey’s multiple comparisons: DMSO vs. BafA1 *p* = 0.151, DMSO vs. Etoposide *p* = 0.0007, BafA1 vs. Etoposide *p* = 0.0074 (**c**, 24 h); *F* = 53.24, *p* < 0.0001 & Tukey’s multiple comparisons: DMSO vs. BafA1 *p* = 0.8011, DMSO vs. Etoposide *p* < 0.0001, BafA1 vs. Etoposide *p* < 0.0001 (**c**, 48 h); *F* = 14.52, *p* = 0.0022 & Tukey’s multiple comparisons: DMSO vs. BafA1 *p* = 0.0017, DMSO vs. Etoposide *p* = 0.1727, BafA1 vs. Etoposide *p* = 0.0431 (**d**, 24 h); *F* = 33.39, *p* < 0.0001 & Tukey’s multiple comparisons: DMSO vs. BafA1 *p* < 0.0001, DMSO vs. Etoposide *p* = 0.1977, BafA1 vs. Etoposide *p* = 0.0006 (**d**, 48 h); *F* = 84.16, *p* < 0.0001 & Tukey’s multiple comparisons: DMSO vs. BafA1 *p* = 0.0505, DMSO vs. Etoposide *p* < 0.0001, BafA1 vs. Etoposide *p* < 0.0001 (**e**, 24 h); *F* = 134.7, *p* < 0.0001 & Tukey’s multiple comparisons: DMSO vs. BafA1 *p* = 0.0116, DMSO vs. Etoposide *p* < 0.0001, BafA1 vs. Etoposide *p* < 0.0001 (**b**, 48 h); *F* = 58.12, *p* < 0.0001 & Dunnett’s multiple comparisons: 0 vs. 24 *p* = 0.7327, 0 vs. 48 *p* < 0.0001 (**h**, TUNEL); *F* = 24.73, *p* = 0.0007 & Dunnett’s multiple comparisons: 0 vs. 24 *p* = 0.389, 0 vs. 48 *p* = 0.0006 (**h**, TUNEL); *t* = 7.503, *p* = 0.0017; *F* = 167.6, *p* < 0.0001 & Tukey’s multiple comparisons: DMSO vs. BafA1 *p* = 0.0296, DMSO vs. Etoposide *p* < 0.0001, BafA1 vs. Etoposide *p* < 0.0001 (**l**); row factor (time) *F* = 0.3245, *p* = 0.626, column factor (treatment) *F* = 5.059, *p* = 0.0337 & Tukey’s multiple comparisons: Untreated vs. BafA1 (24 h) *p* = 0.0239, Untreated vs. BafA1 (48 h) *p* = 0.0077, Untreated vs. BafA1 (72 h) *p* = 0.0273, 24 h vs. 48 h (BafA1) *p* = 0.0334. Raw data and further statistical information provided in the Source Data File and at dois 10.5281/zenodo.18772707, 10.5281/zenodo.18806148, 10.5281/zenodo.19734917 and 10.5281/zenodo.19743163.

Autophagy of the nuclear lamina has been associated with DNA damage and oncogene-induced senescence^20,21,25^. Indeed, the observed LaminB1 nuclear degeneration in our model of proteotoxic stress in neuroblastoma cells is accompanied by an overall reduction in total cell levels of LaminB1 (Fig. 3a, b) and is associated with nuclear γH2AX foci and positive TUNEL staining, indicative of DNA damage (Fig. 3f, g). However, a time course analysis in neuroblastoma cells treated with BafA1 shows that key parameters of LaminB1 nuclear lamina damage all occur prior to DNA damage, which is increased later at 48 h (Fig. 3h). DNA damage is therefore a downstream effect of nuclear lamina damage observed following chronic autophagic clearance impairment. In agreement with this, chemically induced DNA damage via etoposide (Supplementary Fig. 2a, b)—known to induce senescence^26^—is not sufficient to cause LaminB1 cytoplasmic puncta accumulation, nuclear shape irregularity or proteotoxic stress measured by p62 accumulation (Supplementary Fig. 2c–f).

Next, to confirm this divergence in the context of proteotoxic stress induced LaminB1 degeneration, we investigated some of the signalling pathways known to regulate DNA damage response and senescence. Whole cell lysate from neuroblastoma cells subjected to 24 h of autophagic clearance impairment reveals that, while the ATM protein is unaffected at this stage, a significant increase in phosphorylation of the stress sensor p53 is detected (Supplementary Fig. 2g, h and Fig. 3i, j). This observation is accompanied by a transient trend towards increased overall levels of p53 and ultimately results in an increase of the p53 target p21 and its nuclear accumulation (Fig. 3a, c, e, k, l). BafA1 treatment induced changes to p53 and p21 are accompanied by an expected increase in p62 levels to confirm protein clearance inhibition and proteotoxic load (Fig. 3a, d). p53 and p21 increase and LaminB1 reduction are alterations typical of the initiation of cell senescence and can be triggered by low dose etoposide treatment (Fig. 3a, c, e, k, l). However, other senescence markers like p16, SA-β-gal and some Senescence Associated Secretory Phenotype markers like IL1a and IL8 are not affected by BafA1 treatment (Supplementary Fig. 2i, j). The signalling responses to etoposide and BafA1 also diverge in the elevation of p62, indicating absence of proteotoxic stress following etoposide treatment (Fig. 3a, d).

Collectively, this analysis of DNA damage and senescence pathways in our models of autophagic clearance impairment singles out proteotoxic load—not DNA damage—as the trigger of LaminB1 alterations and nuclear degeneration. Although the intracellular events we observe following autophagic clearance impairment include DNA damage and some shared similarities with senescence induction, substantial signalling divergence and the occurrence of DNA damage downstream of LaminB1 degeneration leads us to conclude that the latter is a phenomenon distinct from DNA damage-related senescence.

The observed nuclear and cellular degeneration is likely to be incompatible with cell survival. To assess this in absence of confounding cell proliferation, we utilized post-mitotic rat primary cortical neurons. In agreement with our hypothesis, proteotoxic stress induction with BafA1 results in cell loss, degenerating cell morphology and lack of neurite complexity over time compared to controls (Fig. 3m, n). To understand which cell death mechanism may be implicated in LaminB1 nuclear degeneration, we therefore examined known markers and pathways of apoptotic and necrotic cell death. First, we examined the apoptosis pathway and its associated features. In neuroblastoma cells BafA1-induced proteotoxic stress does not result in the formation of pyknotic nuclei typical of apoptosis and is not associated with classic apoptotic markers like caspase-3 activation or caspase-mediated PARP-1 cleavage^27,28^ (Supplementary Fig. 3a–c). Consistently, the use of a caspase inhibitor does not modify the nuclear abnormalities observed (Supplementary Fig. 3d, e). Collectively, this evidence rules out apoptosis as the mechanism responsible for cell death.

Next, we examined necrotic forms of cell death. Firstly, the cell shrinkage detected following autophagic clearance impairment (Fig. 1b and Supplementary Fig. 1d) is inconsistent with the morphology of necrotic types of cell death^29^. We also observed that markers of secondary necrosis, such as release of lactate dehydrogenase (LDH) into the medium from plasma membrane rupture, are absent (Supplementary Fig. 3f). Furthermore, specific markers of regulated forms of necrosis—such as phospho-MLKL^30^ (necroptosis) and lipid peroxidation^31^ (ferroptosis) (Supplementary Fig. 3g–j)—are not upregulated following treatment with BafA1, displaying if anything a downward trend with respect to the control condition. Additionally, the parthanatos and PARP-1 inhibitor, 3AB, failed to rescue alterations of the nuclear lamina caused by BafA1 treatment (Supplementary Fig. 3k, l), suggesting this does not involve parthanatos. Collectively, the cellular phenotypes observed indicate that proteotoxic stress induced by chronic lysosomal autophagic clearance impairment triggers a type of cell death distinct from other forms^29^ (Supplementary Fig. 3a).

We refer to this type of cell death as “karyoptosis”, since nuclear damage is the first phenotype to emerge^32^. Interestingly, karyoptosis has been independently described in other settings. The cyclic AMP-responsive element binding protein 3 (CREB3) has been separately identified as an inducer of DNA damage and karyoptotic cell death in melanoma cells^33^. Overexpression of the carboxy terminal fragment, CREB3-CF, is also capable of inducing karyoptosis in neuroblastoma cells, while this effect is reduced by multiple inactivating K to R mutations in its bZIP domain (Supplementary Fig. 4a–c). To establish whether CREB3 mediates the karyoptotic pathway triggered by autophagic lysosomal clearance impairment, we employed CREB3 downregulation with shRNA (Supplementary Fig. 4d). However, this does not appear to block LaminB1 nuclear degeneration triggered by BafA1 (Supplementary Fig. 4e, f), indicating that CREB3 does not mediate karyoptosis arising from proteotoxic stress and that multiple independent processes and pathways can instigate karyoptotic cell death.

### The p38 mitogen activated kinase pathway regulates karyoptosis via control of LaminB1 stability

**Fig. 4:**
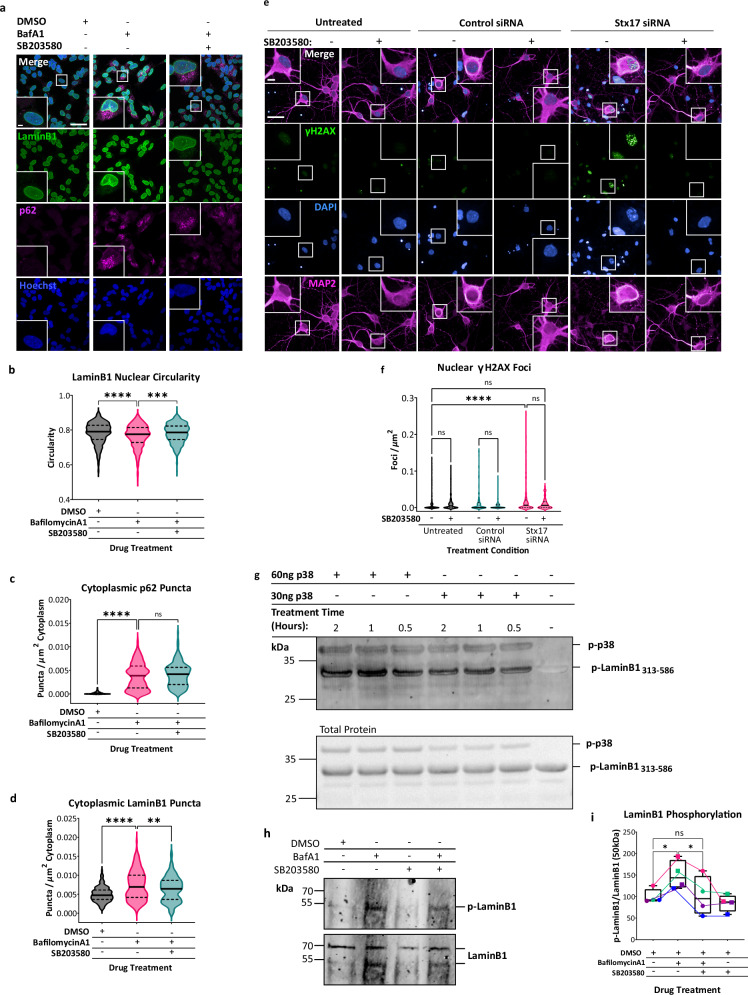
LaminB1 phosphorylation by p38 MAPK regulates LaminB1 nuclear stability in karyoptotic nuclear integrity loss. **a** Images of LaminB1 and p62 immunofluorescence in SH-SY5Y cells treated with BafA1 ± p38 inhibitor SB203580. White box = zoom. Quantification of LaminB1 nuclear circularity (**b**), cytoplasmic p62 puncta (**c**) and LaminB1 cytoplasmic puncta (**d**). **e** Images of γH2AX, DAPI and MAP2 immunofluorescence in rat primary cortical neurons treated with either control siRNA or Syntaxin17 siRNA for 72 h. **f** Quantification of nuclear γH2AX foci. **g** In vitro kinase assay with p38 (30 or 60 ng) and recombinant purified C-terminal (313–586) of LaminB1. Top: Pro-Q Diamond phospho-protein staining, bottom: ponceau total protein. **h** Western blots of phospho-LaminB1 and LaminB1 after immunoprecipitation of LaminB1 in nuclear lysate of SH-SY5Y treated with BafA1 ± p38 inhibitor SB203580. **i** Quantification of phosphorylation of LaminB1 50 kDa nuclear fragment. Scale bars: 50 µm (**a**, **e**), 10 µm (insets in **e**), 5 µm (insets in **a**). Data presented as mean *± *SEM (**i**). Violin plot solid lines represent median, dashed lines interquartile range (**b–d, f**). Box plots denote median and interquartile range, whiskers minimum and maximum values (**i**). Statistics: Kruskal–Wallis test and Dunn’s post-hoc (**f**); one-way ANOVA and Šídák’s post-hoc (**b–d**) two-way ANOVA and Tukey’s post-hoc (**i**); * *p* < 0.05, ** *p* < 0.01, *** *p* < 0.001, **** *p* < 0.0001. *N* (cells per condition): **b** = 434; **c**, **d** = 173; **e**: Untreated/− 186, Untreated/+ 131, Control/− 119, Control/+ 112, Stx17/− 205, Stx17/+ 56; *N* (experiments): **i** = 4, coloured line connects samples from the same experiment, highlighting trend consistencies. *F* = 9.531, *p* < 0.0001 & Šídák’s multiple comparisons: DMSO vs. BafA1 *p* < 0.0001, BafA1 vs. BafA1 + SB203580 *p* = 0.0009 (**b**); *F* = 220.7, *p* < 0.0001 & Šídák’s multiple comparisons: DMSO vs. BafA1 *p* < 0.0001, BafA1 vs. BafA1 + SB203580 *p* = 0.2365 (**c**); *F* = 27.43, *p* < 0.0001 & Šídák’s multiple comparisons: DMSO vs. BafA1 *p* < 0.0001, BafA1 vs. BafA1 + SB203580 *p* = 0.0043 (**d**); Kruskal–Wallis statistic = 26.61, *p* < 0.0001 & Dunn’s multiple comparisons: Untreated/− vs. Untreated/+ *p* = 0.1542, Control/− vs. Control/+ *p* = 0.2874, Stx17/− vs. Stx17/+ *p* > 0.9999, Untreated/− vs. Stx17/− *p* < 0.0001, Untreated/− vs. Stx17/+ *p* = 0.1557 (**f**); row factor (conditions) *F* (3, 9) = 7.750, *p* = 0.0073, column factor (replicates) *F* (3, 9) = 8.843, *p* = 0.0048 & Tukey’s multiple comparisons: DMSO vs. BafA1 *p* = 0.0121, BafA1 vs. SB203580 + BafA1 *p* = 0.0135, DMSO vs. SB203580 + BafA1 *p* = 0.9943 (**i**). Raw data and further statistical information provided in the Source Data File and at dois 10.5281/zenodo.19348450 and 10.5281/zenodo.19709429.

The p38 mitogen activated kinases (MAPK) form a regulatory intracellular pathway capable of orchestrating responses to cell stress, including proteotoxic stress, autophagy, DNA damage and senescence^34^. In neuroblastoma cells, pharmacological inhibition of p38 MAPK reduced cytoplasmic LaminB1 puncta accumulation and nuclear shape irregularity caused by lysosomal autophagic clearance impairment with BafA1 treatment; and reduced hallmarks of DNA damage and cell death in primary rodent neurons with *STX17* knockdown (Fig. 4a–f). Reduction of these features occurs without affecting cytoplasmic p62 accumulation (Fig. 4c), thus uncoupling proteotoxic stress and nuclear lamina pathology and suggesting that p38 MAPK activity is necessary to relay the signal that causes lamina irregularity upon proteotoxic stress. Importantly, the p38α isoform is known to directly phosphorylate LaminB1^35^ and through phosphoproteomic analysis we identified Ser 391 as a p38α target residue on LaminB1 (Supplementary Data 2, Fig. 4g, and Supplementary Fig. 5a). To understand the dynamics of LaminB1 phosphorylation by p38 MAPK in karyoptosis we raised a monoclonal antibody against the p38 MAPK consensus at Ser 391 (P-LamB1^S391^), which specifically recognises in vitro phosphorylated LaminB1 (Supplementary Fig. 5b). After LaminB1 immunoprecipitation, our P-LamB1^S391^ antibody recognises a 70 kDa band and, to a greater extent, a 50 kDa LaminB1 fragment (Supplementary Fig. 5c), similar to the one previously reported^18^. The phosphorylation of the 50 kDa LaminB1 fragment is upregulated during karyoptosis induced by BafA1, and this is rescued by p38 MAPK inhibition (Fig. 4h, i). Therefore, phosphorylation of fragmented LaminB1 by p38 MAPK correlates with karyoptosis.

**Fig. 5:**
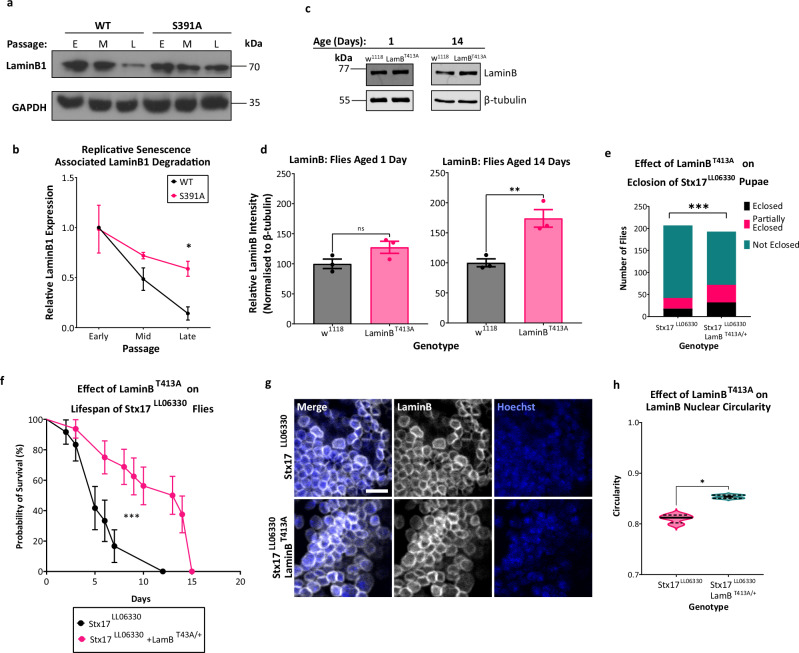
Mutations impairing LaminB1 phosphorylation by p38 MAPK increases protein stability and rescue karyoptosis phenotypes. **a** Western blot in wildtype or mCherry-GFP-LaminB1^S391A^ phospho-mutant human fibroblasts, probing LaminB1 at increasing passage (E early, M mid, L late). GAPDH = loading control. **b** Quantified LaminB1 protein levels from Western blot in human fibroblasts at increasing passage number. **c** Western blots of *Drosophila* LaminB protein levels in whole heads of young (1 day) and aged (14 days) *w*^*1118*^ (wildtype) or heterozygous knock-in *LaminB*^*T413A*^ non-phosphorylatable mutant flies. β-tubulin = loading control. **d** Quantification of relative LaminB levels in 1 day and aged 14 day *w*^*1118*^ wildtype and *LaminB*^*T413A*^ mutant flies. **e** Quantification of pupa viability determined by successful eclosion in *Syntaxin17*^*LL06330*^ mutant *Drosophila *± phospho-mutant *LaminB*^*T413A*^ co-expression. **f** Survival analysis of *Syntaxin17*^*LL06330*^ mutant *Drosophila *± phospho-mutant *LaminB*^*T413A*^ co-expression. **g** LaminB immunofluorescence images in adult brain of *Syntaxin17*^*LL06330*^ mutant *Drosophila* ± co-expression of stabilizing phospho-mutant *LaminB*^*T413A*^. **h** Quantification of LaminB nuclear circularity. Scale bars: 5 µm (**g**). Data presented as mean *± *SEM (**b**, **d**, **f**). In violin plot solid lines represent median, dashed lines interquartile range (**h**). Statistics: two-tailed unpaired *t*-test (**b**, **d**), *χ*^2^ test (**e**), Gehan–Breslow–Wilcoxon test (**f**), Mann–Whitney test (**h**). * *p* < 0.05, ** *p* < 0.01, *** *p* < 0.001. *N* = 3 independent experiments (**b**, **i**); pupae: Stx17^LL06330^ = 207, Stx17^LL06330^ + LamB^T413A/+^ = 193 (**e**); flies: Stx17^LL06330^ = 12, Stx17^LL06330^ + LamB^T413A/+^ = 16 (**f**); 4 per group (**h**). Tukey’s multiple comparisons test: Early WT vs S391A *p* = 0.9246, Mid WT vs S391A *p* = 0.179, Late WT vs S391A *p* = 0.0188 (**b**). 1 day flies: *t* = 2.153, df = 4, *p* = 0.0976; 14 day flies: *t* = 4.624, df = 4, *p* = 0.0098 (**d**). *Χ*^2^ = 14.22, df = 2, *p* = 0.0008 (**e**). Gehan–Breslow–Wilcoxon test *Χ*^2^ = 10.92, df = 1, *p* = 0.001 (**f**). Mann–Whitney *U* = 0, *p* = 0.0286 (**h**). Raw data and further statistical information provided in the Source Data File and at 10.5281/zenodo.19349021.

To assess the functional role of this post-translational modification in the context of karyoptosis and LaminB1 stability, we generated a non-phosphorylatable S391A LaminB1 mutant. When expressed in fibroblasts, LaminB1^S391A^ demonstrates resistance to LaminB1 protein level reduction associated with replicative senescence (Fig. 5a, b). To address whether this enhanced LaminB1 nuclear stability impacts LaminB1 dynamics in aging and karyoptosis at a whole-organism in vivo level, we generated a non-phosphorylatable T413A *LaminB* (*Lam*) CRISPR isogenic knock-in *Drosophila* mutant model. The engineered mutation targets the equivalent p38 consensus sequence in the single *Drosophila* B-type Lamin, *LaminB*, a homologue of mammalian LaminB1. When heterozygous, the *Lam*^*T413A*^ allele had no visible effect on fly morphology, lifespan or neuronal nuclear lamina morphology. However, when aged for 2 weeks at 29 °C, heterozygous *Lam*^*T413A*^ *Drosophila* demonstrate elevated LaminB expression levels compared to control flies, which demonstrate typical age-related LaminB decline (Fig. 5c, d). This suggests a role for phosphorylation in LaminB stability in aging.

To assess the effect of Lam^T413A^ in the context of an autophagic clearance impairment model, next we utilised homozygous mutant *Stx17*^36^ *Drosophila* as a genetic approach similar to our previously used *Stx17* knockdown in vitro. Homozygous mutant *Stx17*^36^ almost eliminates adult fly viability, induces visible accumulation of the *Drosophila* p62 homologue, Ref(2)P, and displays karyoptotic nuclear shape abnormalities in the few flies that do survive into adulthood (Supplementary Fig. 6a–c). In agreement with cellular data, these observations occur in the absence of apoptotic Dcp1 caspase activation (Supplementary Fig. 6d). Most importantly, when combined with mutant *Stx17*, the *Lam*^*T413A*^ allele ameliorates fly survival deficits after pupal eclosion, extends adult fly lifespan and rescues the karyoptotic neuronal nuclear lamina shape alteration (Fig. 5e–h).

Together, these results demonstrate that LaminB1 stability at the nuclear lamina and its dynamics in karyoptosis are directly controlled by p38 MAPK through direct phosphorylation of LaminB1. Cellular degeneration by karyoptosis, therefore, represents a type of cell death fundamentally driven by the regulation of LaminB1 stability.

### Neurons undergo karyoptosis in in vitro and in vivo models of neurodegenerative disease caused by proteotoxic stressors

To examine the role of karyoptosis in human disease, we have focused on neurodegenerative disorders. Lysosomal autophagic clearance is critical to survival in long-lived neurons, as evidenced in many neurodegenerative diseases^1,18,37,38^. In particular, nuclear dysfunction, proteotoxic stress and lysosomal autophagic clearance impairment are prominent features of neurodegenerative ALS/FTD *C9ORF72* repeat expansion mutant models^39,40^. In *C9ORF72* ALS/FTD, dipeptide repeat proteins (DPRs) translated from the repeat expansion mutation produce pathological inclusions capable of impacting lysosomal autophagic clearance, which are also known to pathologically alter the function of other nuclear membrane components, such as nuclear pore complexes^41–48^.

**Fig. 6:**
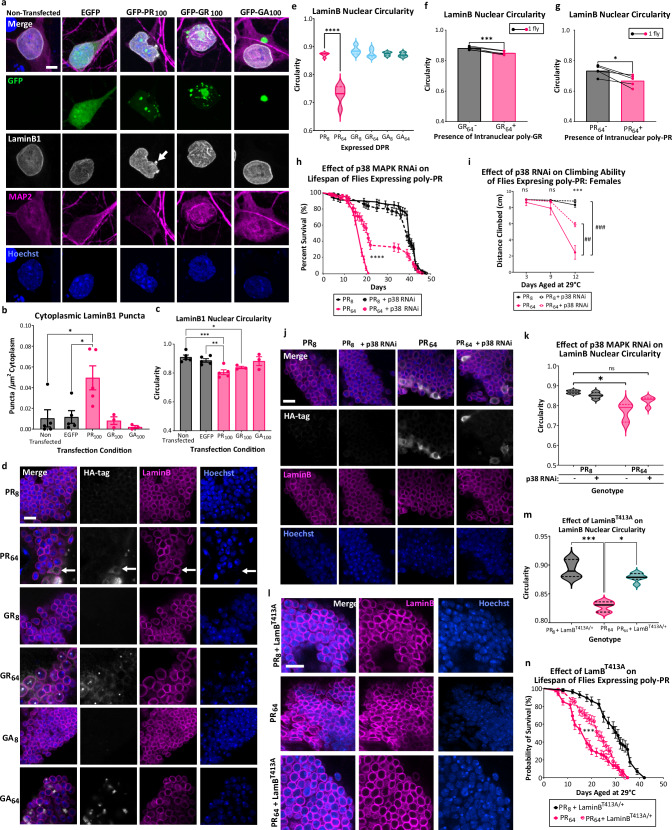
Suppressing LaminB1 phosphorylation by p38 MAPK rescues karyoptosis triggered by *C9ORF72* DPRs in in vitro and in vivo ALS/FTD models. **a** Images of rat primary cortical neurons expressing 100 repeat length GFP-tagged *C9ORF72* DPRs with immunofluorescence probing LaminB1, MAP2 and Hoechst. Arrow: example of cytoplasmic LaminB1 puncta in neuron expressing PR_100_. Quantification of (**b**) LaminB1 cytoplasmic puncta and (**c**) LaminB1 nuclear circularity in primary cortical neurons not transfected or expressing EGFP or EGFP-DPRs. **d** Immunofluorescence of adult *Drosophila* expressing 8-repeat length or 64-repeat length HA-tagged non-HRE PR, GR or GA in the brain. **e** Quantification of LaminB nuclear circularity. **f**, **g** Quantification of LaminB nuclear circularity in *Drosophila* brain expressing PR_64_ (**f**) or GR_64_ (**g**) comparing cells with and without visible intranuclear DPR inclusions. **h** Survival analysis of female *Drosophila* co-expressing either PR_8_ or PR_64_ with p38 MAPK RNAi (52277GD) in the adult brain. **i** Climbing motor function analysis in female *Drosophila* co-expressing either PR_8_ or PR_64_ with p38 MAPK RNAi (52277GD) in the adult brain. **j** LaminB and HA-tagged DPR immunofluorescence images from female adult *Drosophila* brain expressing either PR_8_ or PR_64_ ± p38 MAPK RNAi. **k** Quantification of LaminB nuclear circularity. **l** Immunofluorescence images of LaminB and HA-tagged DPRs in adult *Drosophila* expressing either PR_8_ or PR_64_ ± phospho-mutant LaminB^T413A^ in the brain. **m** Quantification of LaminB nuclear circularity. **n** Survival analysis of *Drosophila* expressing either PR_8_ or PR_64_ ± phospho-mutant LaminB^T413A^ in the adult brain. Scale bars: 5 µm (**a**, **d**, **j**), 10 µm (**l**). Data presented as mean *± *SEM (**b**, **c**, **f–i**, **n**). In violin plots solid lines represent median, dashed lines interquartile range (**e**, **k**, **m**). Statistics: one-way ANOVA followed by post-hoc (**b**, **c**, **i**); Kruskal–Wallis test and Dunn’s post-hoc (**e**, **k**, **m**); unpaired *t*-test (**f**, **g**); Kaplan Meyer analysis with Gehan–Breslow–Wilcoxon test (**h**, **n**). * *p* < 0.05, **/## *p* < 0.01, ***/### *p* < 0.001, ****/#### *p* < 0.0001. # denote post-hoc comparisons within each time point (**i**). *N* = individual experiments (**b**, **c**: 5); flies (**e**: PR_8_ *n* = 5, PR_64_ *n* = 5, GR_8_ *n* = 4, GR_64_ *n* = 5, GA_8_ *n* = 4, GA_64_ *n* = 3; **h** 80 per group; **i** 18 per group, 2 experimental replicates; **m** 6 per group; **n** PR_8_ + *LamB*^*T413A*^ *n* = 57, PR_64_ + *LamB*^*T413A*^ *n* = 87). *F* = 5.313, *p* = 0.0064 and Šídák’s multiple comparisons tests: non-transfected vs PR_100_ *p* = 0.011, EGFP vs PR_100_ *p* = 0.0139, non-transfected vs GR_100_ *p* = 0.9996, non-transfected vs GA_100_ *p* = 0.9479 (**b**). *F* = 0.4603, *p* = 0.0021 and Šídák’s multiple comparisons tests: non-transfected vs PR_100_ *p* = 0.0009, EGFP vs PR_100_ *p* = 0.0077, non-transfected vs GR_100_ *p* = 0.0495, non-transfected vs GA_100_ *p* = 0.7498 (**c**). *F* = 53.54, *p* < 0.0001 and Bonferroni multiple comparisons PR_8_ vs PR_64_ *p* < 0.0001, GR_8_ vs GR_64_ *p* > 0.9999, GA_8_ vs GA_64_ *p* > 0.9999 (**e**). *t* = 9.254, df = 4, *p* = 0.0008 (**f**). *t* = 2.965, df = 5, *p* = 0.0313 (**g**). Gehan–Breslow–Wilcoxon test *Χ*^2^ = 164, df = 3, *p* < 0.0001 (**h**). Day 3: *F* = 1.373, *p* = 0,3716; Day 9: *F* = 2.474, *p* = 0.2011; Day 12: *F* = 75.53, *p* = 0.0006 and Bonferroni’s multiple comparisons: PR_8_ vs PR_64_ *p* = 0.001, PR8 vs PR_8_ + p38 RNAi *p* > 0.9999, PR_64_ vs PR_64_ + p38 *p* = 0.0075, PR_8_ vs PR_64_ + p38 RNAi *p* = 0.0276 (**i**). *F* = 6.448, *p *= 0.0127 and Tukey’s multiple comparisons: PR8 vs PR_8_ + p38 RNAi *p* = 0.8929 PR_8_ vs PR_64_ *p* = 0.0182, PR_8_ vs PR_64_ + p38 RNAi *p* = 0.303, PR_8_ + p38 RNAi vs PR_64_ *p* = 0.0198, PR_8_ + p38 RNAi vs PR_64_ + p38 RNAi *p* = 0.5115, PR_64_ vs PR_64_ + p38 RNAi *p* = 0.1433 (**k**). Kruskal–Wallis statistic = 12.78, *p* < 0.0001 and Dunn’s multiple comparisons: PR_8_ + LamB^T413A^ vs PR_64_ *p* = 0.0009, PR_64_ vs PR_64_ + LamB^T413A^ *p* = 0.0401 (**m**). Gehan–Breslow–Wilcoxon test *Χ*^2^ = 11.51, df = 1, *p* = 0.0007 (**n**). Raw data and further statistical information provided in the Source Data File and at 10.5281/zenodo.19353588.

To test whether proteotoxic stress generated by the *C9ORF72* DPRs induces karyoptosis, we utilised rat primary cortical neurons, which we previously validated as a model for karyoptotic cell death induced by BafA1 (Fig. 3m, n). Rat primary cortical neurons expressing *C9ORF72* DPRs display features consistent with karyoptosis. In particular, the proline-arginine (poly-PR) DPR strongly induced LaminB1 cytoplasmic puncta, nuclear shape abnormalities and TUNEL-positive DNA damage indicative of cell death, again, in the absence of apoptotic caspase-3 activation (Fig. 6a–c and Supplementary Fig. 7a–c). Expression of glycine-arginine (poly-GR) DPR was also associated with some of these features, albeit to a reduced extent, while we did not detect any karyoptotic effect associated with the glycine-alanine (poly-GA) DPR (Fig. 6a–c).

*Drosophila melanogaster* has been widely used to model *C9ORF72* DPR toxicity in vivo^44,45,47–50^. A model of inducible DPR expression^47^ in adult *Drosophila* brain recapitulates our observations seen in rodent primary cortical neurons, whereby PR_64_ flies demonstrate LaminB nuclear shape alterations and TUNEL-positive DNA damage (Fig. 6d, e and Supplementary Fig. 7d). Consistently, these features are observed in the absence of apoptotic markers like cleaved caspase-3 or Dcp1 (Supplementary Fig. 7e, f). Furthermore, caspase inhibition with a dominant negative *Dronc* transgene^51^ does not affect the severe PR_64_-induced lifespan reduction (Supplementary Fig. 7g). The autophagy-lysosome system is challenged in PR_64_ and GR_64_ flies, causing widespread proteotoxic stress, demonstrated by brain accumulation of Ref(2)P (Supplementary Fig. 8a, b). Importantly, cells displaying PR_64_ or GR_64_ intranuclear inclusions show more dramatic LaminB nuclear shape distortion than nuclei within the same fly which do not contain detectable inclusions (Fig. 6f, g). This therefore indicates a direct link between DPR protein aggregation levels, proteotoxic stress load and karyoptotic lamina degeneration. These results agree with our earlier observations in cells and flies, where proteotoxic stress was produced as a secondary consequence of pharmacological or genetic impairment of lysosomal autophagic clearance, and suggest that the mechanism of cell death identified with those tools may be relevant to human neurodegenerative diseases associated with proteotoxic stress.

To test this hypothesis, we have exploited the severe shortening of the fly lifespan and progressive motor impairment caused by PR_64_ and GR_64_ to assess potential therapeutic relevance of p38 MAPK regulation of LaminB in vivo. Importantly, motor impairment and reduced lifespan of PR_64_ or GR_64_ flies are partially rescued when either *p38a* or *p38β* isoforms are knocked down, and LaminB nuclear shape is also no longer significantly altered (Fig. 6h–k and Supplementary Fig. 8c–f). Additionally, to gain direct insight into the importance of LaminB regulation via its phosphorylation by p38 MAPK, we have combined the *Lam*^*T413A*^ allele with PR_64_ flies. This results in a significant rescue of the neuronal nuclear lamina shape abnormalities and of the fly lifespan (Fig. 6l–n).

**Fig. 7:**
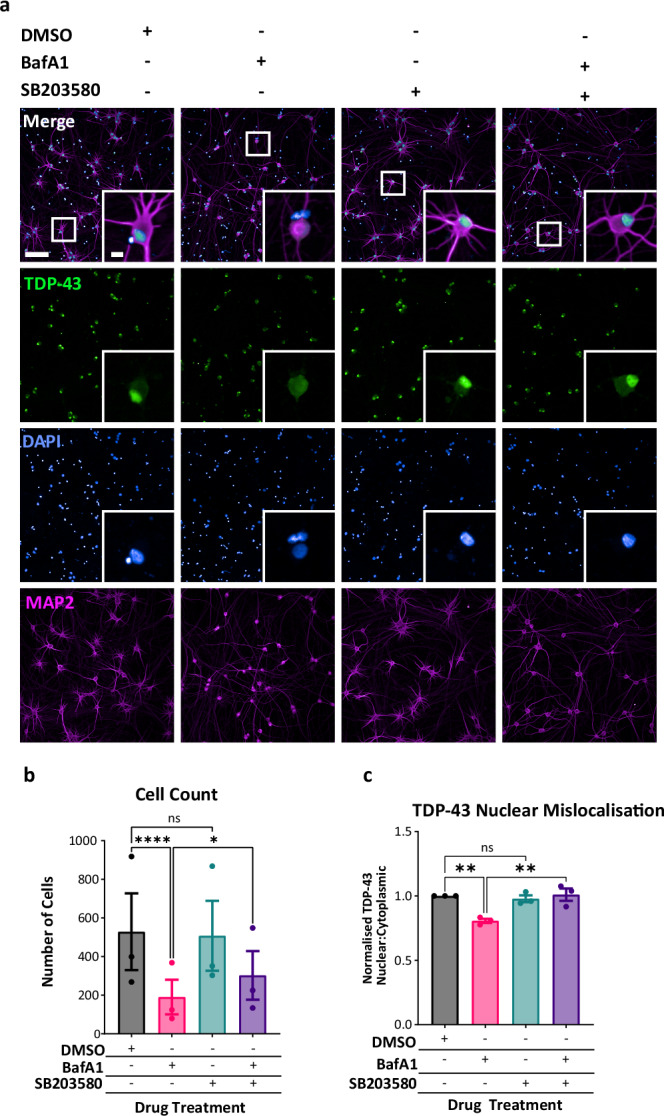
Neurodegenerative features in human neurons are affected by p38 MAPK-dependent karyoptosis. **a** Images of TDP-43 C-terminal, MAP2 and DAPI immunofluorescence in hiPSC-derived cortical neurons treated with DMSO, BafA1 ± p38 MAPK inhibitor SB203580 at DIV21. **b** Quantification of MAP2 positive neuron cell count. **c** Quantification of nuclear:cytoplasmic ratio of TDP-43 fluorescence mean intensity. Scale bars: 50 µm (**a**) and 10 µm (**a** insets). Data presented as mean *± *SEM (**b**, **c**). Statistics: two-way ANOVA followed by Tukey’s post-hoc multiple comparisons (**b**, **c**). Asterisks denote significance of post-hoc comparisons. * *p* < 0.05, ** *p* < 0.01, *** *p* < 0.001, **** *p* < 0.0001. *N* = 3 experimental replicates. *F* = 125.1, % of total variance = 63.28, *p* < 0.0001 (experimental replicate) and *F* = 38.4, % of total variance = 29.13 and *p* < 0.0001 (drug treatment). Tukey’s post-hoc comparisons: DMSO vs BafA1 *p* < 0.0001; DMSO vs SB203580 *p* = 0.9405; BafA1 vs BafA1 + SB203580 *p* = 0.0265 (**b**). *F* = 4.192, % of total variance = 11.26 and *p* = 0.0726 (experimental replicate) and *F* = 20.02, % of total variance = 80.68 and *p* = 0.0016 (drug treatment. Tukey’s post-hoc comparisons: DMSO vs BafA1 *p* = 0.0028; DMSO vs SB203580 *p* = 0.8829; BafA1 vs BafA1 + SB203580 *p* = 0.0022 (**c**). Raw data and further statistical information provided in the Source Data File and at 10.5281/zenodo.20274655.

Finally, to establish whether karyoptosis is associated with neuronal pathology in a human neuronal model, we treated human neurons derived from engineered KOLF2.1J wildtype hNGN2 iPSC (Supplementary Fig. 9a–d) with BafA1 for 24 h. We detect a significant reduction in cell number and a decrease in TDP-43 nucleo/cytoplasmic ratio, an important pathological hallmark of ALS/FTD^52^. Crucially, both parameters are significantly rescued by p38 MAPK inhibition (Fig. 7a–c), validating the relevance of this pathway for ALS/FTD pathology in a human neuronal model.

Taken together, these results provide evidence for a direct link between levels of proteotoxic stress and karyoptosis in neuronal cell cultures and in-vivo models of ALS/FTD proteotoxicity and its regulation by the p38 MAPK pathway through LaminB1 phosphorylation.

### Karyoptosis hallmarks are present in neurodegenerative disease patient brain tissue

Finally, to validate and quantify the impact of karyoptosis in human neurodegenerative disease, we examined LaminB1 immunofluorescence in *post-mortem* frontal cortex from dementia patients exhibiting frontotemporal lobar degeneration (FTLD) or Alzheimer’s disease (AD) pathology, and age and sex matched controls (Supplementary Data 3). To identify whether populations of cells with karyoptotic hallmarks could be detected, we assessed LaminB1 nuclear circularity along with cytoplasm and nuclear area as measures of atrophy, applying the k-means clustering algorithm to single-cell immunofluorescence data^53,54^. This is an unsupervised machine learning technique commonly used to identify groups of similar data points in large data sets in an unbiased manner. Importantly, the algorithm was blind to which neuropathological classification group (control, FTLD or AD) each cell originated from. In the first and largest dataset tested, we identified four different neuronal phenotype clusters in deep layers of the frontal cortex (Supplementary Fig. 10a–d). Cluster 4 emerges as a group containing cells likely to be undergoing karyoptosis based on low LaminB1 nuclear circularity and small cytoplasmic area suggestive of atrophy, relative to the other clusters (Supplementary Fig. 10d). When the neuropathological classification group identities of the neurons sorted into this cluster are revealed and relative proportions compared, neurons originating from both FTLD and AD (Braak stage 6) cases are significantly overrepresented (Supplementary Fig. 10a, e). Conversely, it is possible to identify a clear control phenotype group—Cluster 1—whose defining properties are a relatively large cytoplasm area and regular LaminB1 circularity, and within which, neurons from healthy control cases are overrepresented (Supplementary Fig. 10a, d, e).

**Fig. 8:**
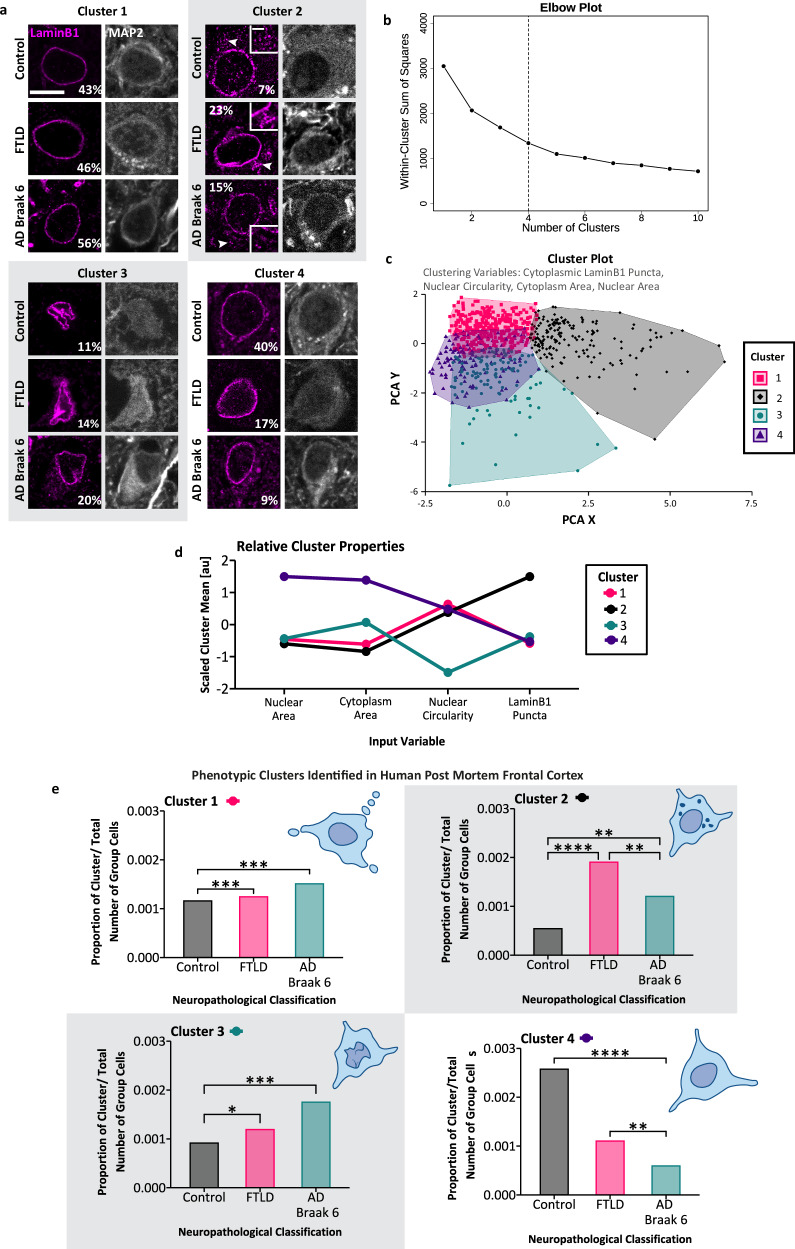
Karyoptotic hallmarks are over-represented in *post-mortem* FTLD and AD human frontal cortex. **a** Examples of LaminB1 and MAP2 immunofluorescence in single cells from each neuropathological classification group found within each phenotype cluster identified by k-means clustering. % = number of cells within the cluster from each group (control, FTLD, AD) shown as a percentage of the total sample for each group (control, FTLD, AD). **b** Elbow plot demonstrating within clusters sum of squares method to model the most appropriate value of *k* to use in k-means cluster analysis for each data set. Vertical dotted line indicates the determined “elbow” point of the plot, at *k* = 4. **c** Principal component plot illustrating clusters fitted to data sets by k-means cluster analysis. **d** Parallel coordinates plot visualizing relative differences in cluster mean for each input variable across all clusters identified by k-mean cluster analysis of single neuron LaminB1 immunofluorescence in *post-mortem* brain. **e** Frequency bar charts indicating the proportion of neurons from each neuropathological classification group (control, FTLD, AD) within each identified cluster. Proportion is normalised according to overall total number of cells sampled from each neuropathological classification group. * *p* < 0.05, ** *p* < 0.01, *** *p* < 0.001, **** *p* < 0.0001. The grey shaded boxes identify the two clusters with morphology compatible with karyoptosis at different stages (**a** and **e**). Scale bars: 50 µm (**a**), 10 µm (**a** insets). Statistics: *χ*^2^ test followed by pairwise post-hoc binomial test with Bonferroni correction (**f**). Immunofluorescence dataset of 763 cells, from 20 separate cases (Control: *n* = 7, FTLD: *n* = 7, AD: *n* = 6). Cluster 1: *χ*^2^ = 18.28, *p* = 0.0001073; and pairwise post-hoc binomial tests: Control vs FTLD *p* = 0.000299, Control vs AD *p* = 0.000072, FTLD vs AD *p* = 0.7676. Cluster 2: *χ*^2^ = 40.479, *p* = 1.62e^−9^; and pairwise post-hoc binomial tests: Control vs FTLD *p* = 1.33e^−10^, Control vs AD *p* = 0.00062, FTLD vs AD *p* = 0.00269. Cluster 3: *χ*^2^ = 13.672, *p* = 0.001074; and pairwise post-hoc binomial tests: Control vs FTLD *p* = 0.01114, Control vs AD *p* = 0.000256, FTLD vs AD *p* = 0.3049. Cluster 4: *χ*^2^ = 29.277, *p* = 4.39e^−7^; and pairwise post-hoc binomial tests: Control vs FTLD *p* = 0.2274, Control vs AD *p* = 4.98e^−8^, FTLD vs AD *p* = 0.00176. Raw data and further statistical information provided in the Source Data File and at 10.5281/zenodo.19768610.

To confirm the discovery and gain further insight regarding other phenotype clusters, we implemented a new analysis of most cases in the same cohort, where, thanks to improved autofluorescence quenching, measurement of cytoplasmic LaminB1 puncta could be incorporated as an additional input variable (Fig. 8a–d). As before, four distinct phenotype clusters are identified, including two clusters that share the same characteristics as in the previous analysis: a control cluster (Cluster 4) and a cluster likely to include cells at the late stage of karyoptosis (Cluster 3) on the basis of reduced LaminB1 nuclear circularity. Therefore, the second dataset provides a confirmation of the reproducibility of k-means identification of karyoptotic and control clusters of neurons. In addition to this, incorporation of cytoplasmic LaminB1 puncta as an input variable highlights a new interesting cell group, Cluster 2, distinguished by a relatively high density of cytoplasmic LaminB1 puncta and low cytoplasm area, in which neurons from FTLD cases, and to a lesser extent AD cases, are overrepresented (Fig. 8e). Cluster 2 includes neurons consistent with the features of early stage karyoptosis as defined at 12 h in our in vitro time course models (see Fig. 1b), in which LaminB1 puncta accumulate in the cytoplasm but nuclear shape is not yet dramatically altered. The remaining group, Cluster 1, is the largest in terms of cell numbers and is probably composed of a mixture of cell phenotypes. However, it is significantly overrepresented in disease and exhibits low nuclear and cytoplasm area, so is likely to contain degenerating neurons, which do not present obvious karyoptotic features. We therefore speculate that Cluster 1 includes neurons undergoing degeneration via a different cell death pathway. Interestingly, a similar population of neurons, enriched in disease but without karyoptotic features, are found in Cluster 2 in our first dataset (Supplementary Fig. 10d, e and Supplementary Data 3).

Finally, this approach provides a quantitative estimate of the relevance of karyoptosis in neurodegenerative disease. The effect size of early and late-stage karyoptosis in our second dataset found within the karyoptosis clusters, when considering the percentage of neurons from each neuropathological classification group, is 35–37% in FTLD or AD compared to 17% in controls (Fig. 8e and Supplementary Data 3, Clusters 2 and 3). The presence of neurons with karyoptotic features from age-matched cases without a diagnosis of a neurodegenerative disease suggests that this process could also contribute to cell death in normal ageing. However, when considering the additional effect in neurodegenerative disease, karyoptosis appears to be responsible for a remarkable extra 18–20% of cell degeneration in FTLD or AD. Similarly, an extra 8–12% of late stage karyoptotic cell degeneration in FTLD and AD is identified in our first dataset. Thus, karyoptosis could account for a substantial proportion of neuronal degeneration, and ultimately cell death, in the forms of dementia assessed here. This result also highlights the utility of large-scale single-cell phenotyping approaches to identify and measure populations of karyoptotic neurons in patient tissue. In conclusion, unbiased immunofluorescence and morphological phenotyping identifies populations of cells consistent with karyoptosis features in patient brain tissue and emphasizes the significant role of karyoptosis in human disease.

## Discussion

Our study identifies and characterises karyoptosis as a distinct mechanism of cell degeneration and death that can stem from proteotoxic stress exerted by protein aggregates and/or lysosomal autophagic clearance impairment. In karyoptosis, the nuclear lamina degenerates, forming cytoplasmic puncta that are then released in extracellular vesicles. Notwithstanding whether the extracellular vesicles primarily aim to relieve proteotoxic stress or are part of the death process itself, they represent a terminal event. The end result is an overall reduction in intracellular LaminB1 levels and an outflow of nuclear material, culminating in nuclear degeneration, DNA damage, cell atrophy and death. This process is regulated by the p38 MAPK pathway through LaminB1 phosphorylation and can be detected in a substantial proportion of neurons from patient tissue with human neurodegenerative diseases. As we have observed karyoptosis in a range of cells, we predict that it could also be involved in many human disorders where cells experience significant proteotoxic stress caused by diverse genetic, environmental or immunological factors. An intriguing possibility consistent with our data on *C9ORF72* DPRs is that karyoptosis may be particularly connected to the presence of nuclear proteotoxic stressors, which are known to be more efficient in generating irreparable nuclear envelope damage^55^. Proteotoxic stress is however, not the only trigger of karyoptosis, which can arise also through other mechanisms and, for example, has been identified as an important contributor to the death of UV-irradiated melanoma cells regulated by CREB3^33^. Some features of karyoptosis like caspase independence and nuclear deformation, overlap with Linker Cell Death, defined in *C. elegans*^56,57^ and with cell death identified in AD animal models^15,58^, however it is unclear whether they represent the same process and share molecular regulation pathways, thus warranting further investigation.

Our study has primarily identified the mechanism of karyoptosis using in vitro models and while substantial validation has also been conducted in vivo and in *post-mortem* human samples, a complete reconstruction of molecular events and timeline remains to be established. The precise role of the EV shedding in the death process of karyoptosis, and the impact of released EVs on the surrounding tissue also remains to be fully elucidated. Finally, a detailed molecular analysis of nuclear lamina fragmentation and nuclear material loss awaits additional characterization. Notwithstanding these limitations, the identification of karyoptosis has significant implications for dementia and other neurodegenerative diseases. While karyoptosis may be one of many mechanisms involved in addition to apoptosis and regulated necrosis, understanding the quantitative impact of different cell death forms on the neuronal cell loss responsible for clinical symptoms will inform therapeutic targeting in neurodegenerative diseases. Extending cell survival is critical to sufficiently enhance the therapeutic window for interventions targeting upstream causes of disease. Here, we identify p38 MAP kinase and its phosphorylation of LaminB1 as possible targets for neuroprotective treatments that stop or delay karyoptosis and may thus critically extend neuronal survival.

## Methods

### Ethics statement

Human *post-mortem* tissue use is approved under the Health Research Authority REC reference 18/WA/0206. All other methods used do not require ethical approval. The research involves human iPSC lines, derived from the KOLF2.1j background obtained from Jackson Laboratories, which has well-documented origin obtained under informed donor consent and under relevant ethical oversight^59^.

### Plasmids

Alternate codon non-G_4_C_2_ pcDNA3.1(+)-GFP-DPR_100_ plasmids are previously described^60^. To generate an EGFP control plasmid, GFP was extracted from pEGFP-N1 (Clontech, 6085-1) and subcloned into the pcDNA3.1(+) vector using BamHI and NotI restriction sites. pBabe-mCherry-GFP-LaminB1 retroviral vector is previously described in ref. ^20^, from which a pBabe-mCherry-GFP-LaminB1^S391A^ construct was subsequently generated by site-directed mutagenesis. A LaminB1^313–585^ fragment plasmid for bacterial expression was made to generate recombinant purified LaminB1 for in vitro kinase assay use. Chemically synthesised DNA encoding the human LaminB1^313–585^ fragment was subcloned into a pETDuet-1 vector and transformed into *E. coli* Rosetta II cells. To generate a pCLYBL-TO-hNGN2-hSyn-mCherry plasmid, mCherry was amplified from a gene synthesised mCherry cDNA (GeneArt, Thermo Fisher) using mdr1035 (5′-TGTTCTGCGCCGTTACAGATCC-3′) and mdr1036 (5′-TTGATATCACTACTTGTACAGCTCGTCCATG-3′) primers and inserted into EcoRV and NheI digested pCLYBL-TO-hNGN2-BSD-mApple^61^ (kind gift from Michael Ward, Addgene #124229), resulting in pCLYBL-TO-hNGN2-mCherry. Subsequently, the EF1A promoter was replaced with a gene synthesised human synapsin promoter (“hSyn”) (GeneArt, Thermo Fisher) using AsiSI and NheI restriction sites, resulting into pCLYBL-TO-hNGN2-hSyn-mCherry. The pEF1a-CRE-T2A-mCherry plasmid was cloned by inserting a CRE-T2A-mCherry cDNA (GeneArt, Thermo Fisher) into the EcoRI, XbaI sites of pRR-EF1a-R495X^62^.

### Mammalian cell line culture and pharmacological treatments

SH-SY5Y (ATCC, CRL-2266), PtK2 (ATCC, CCL-56) and MRC-5 fibroblast (ATCC, CCL-171) cells were grown in Advanced DMEM/F12 with 10% FBS, 5% penicillin-streptomycin and 5% L-glutamine (Gibco). For immunohistochemical assays, SH-SY5Y cells were grown in coverslip-bottom 96-well plates (ibidi, 89626). For biochemical assays and EV generation cells were grown in 6-well plates and 150 mm dishes, respectively. PtK2 cells were grown in 35 mm glass coverslip bottom dishes (Griener Bio-one).

SH-SY5Y and PtK2 cells were treated with either DMSO (0.1%) or BafilomycinA1 (Sigma) or Leupeptin (Sigma) in DMSO 0.1% at concentrations and time durations indicated in figure legends. In p38 inhibition experiments, SH-SY5Y were treated with 20 µM SB203580 (Tocris Bioscience, 1202). In experiments measuring DNA damage, SH-SY5Y cells were treated with 10 µM etoposide (Sigma, E1383)^26^ as a positive control condition. In experiments probing markers of apoptosis, as a positive control a sample of cells were treated with 1 µM staurosporine (Sigma, S6942) for 4 h prior to the fixation time point. In experiments with inhibition of caspases 10 μM Z-VAD-FMK (Sigma) was added as a pre-treatment 1 h prior to other treatments and maintained for the whole duration of the experiment. To induce ferroptosis, 35 μM 6-Hydroxidopamine (6-OHDA, Sigma) in 0.02% ascorbic acid (AA) was used. The same concentration of AA was used as negative control. To inhibit PARP-1 and parthanatos cells were pretreated with 3-aminobenzamide (3-AB, Sigma) at 500 μM for 1 h, prior to exposure to DMSO or BafA1. Primary human fibroblasts (IMR90) stably expressing tamoxifen-activatable oncogenic H-RasV12 fused to the estrogen receptor (ER) ligand-binding domain. H-Ras expression is induced with tamoxifen which causes cells to undergo oncogene-induced senescence^63^. Cells were grown in DMEM glutamax, 10% FBS, 100 U/ml of penicillin, 100 ug/ml of streptomycin and were cultured in the presence of 100 nM +4OHT- tamoxifen (or EtOH control), passaging when sub-confluent, until senescent at day 12.

### Rodent primary cortical neuron culture

Cortical neurons were isolated from E18 Sprague Dawley rat embryos (Charles River) and cultured in Neurobasal medium containing 0.25% penicillin-streptomycin, 0.25% Glutamax and 2% B-27 (all Gibco), on 13 mm diameter glass coverslips coated in 0.1 mg/ml poly-D-Lysine

### Human KOLF2.1J WT hNGN2 iPSC line generation and iPSC-derived Neuron Culture

KOLF2.1J-WT (JIPSC001000, JAX) iPSCs^59^ with heterozygous insertion of a tetracycline-inducible human Neurogenin-2 (hNGN2) expression cassette in the *CLYBL* locus were generated via electroporation of KOLF2.1J-WT with pCLYBL-TO-hNGN2-hSyn-mCherry vector, a *CLYBL* locus-targeting gRNA (5′-ATGTTGGAAGGATGAGGAAA-3′, IDT) and recombinant Cas9 nuclease (1081060, IDT) using a Nucleofector 2B (Lonza, VPH-5022 kit). At 7 days post-electroporation, positive cells were selected with 200 ug/ul Geneticin (G418, Gibco) for 7 days, after which colonies were picked. Clones were screened for homo/heterozygosity and

correct integration of the cassette by PCR (left homology arm JA16: 5′-GCCACCAGATAACTGTACC-3′ and JA17: 5′-TGTGGGAGGAAGAGAAGAG-3′; right homology arm JA18: 5′-AGTCAATCCGCCGAGTTGG-3′ and JA19: 5′-GCTCAGATGGAGCAGTGGATG-3′; *CLYBL* WT locus: JA16 and JA19). Integrity of the WT *CLYBL* allele in heterozygous clones was assessed by PCR (JA16 and JA19) followed by Sanger sequencing (AP58: 5′-GCAAATTGAATAGGTGAATG-3′ and AP59: 5′-ACAAATCAAACATGGCTCAG-3′). Selected clones were subjected to pluripotency assessment by IF, qPCR and trilineage differentiation embryoid bodies assay. For CRISPR off-target analysis, the top 3 off-target chromosomal regions were identified by IDT online tool (https://www.idtdna.com/site/order/designtool/index/CRISPR_SEQUENCE). Ch17, Ch8 and Ch4 regions were amplified by PCR (Ch17 JA22: 5′-GACACTCCAGCAGCTTCTTG-3′ and JA23: 5′-TTGTCCTGAGGGTGGGAATC-3′; Ch8 JA26: 5′-GTTTGTTCCCTGGTGCATTC-3′ and JA27: 5′-CAGAGACACCCACTTCATTG-3′; Ch4 JA30: 5′-GGAGCCACACTGCCTGATTG-3′ and JA31: 5′-CAGAGCACCACCACGTGTTC-3′) and Sanger sequenced (Ch17 JA24: 5′-GAAAGGGCTGAAGGCAATAC-3′ and JA25: 5′-GAAGGAACTCAGGGCGTGTC-3′; Ch8 JA28: 5′-GCTTTGAAGGCTGCCGTCTG-3′ and JA29: 5′- GGTGCGATGTTGGCTCACTG-3′; Ch4 JA32: 5′-ATTTGACTGCCCTCTGCTTC-3′ and JA33: 5′- GAACTGGATAGGGCATGAGG-3′). Karyotyping was carried out with KaryoStat+ analysis (Thermo Fisher). A fully validated heterozygous clone was taken forward for further editing to remove the mCherry and antibiotic selection cassette.

Excision of the floxed cassette was carried out by electroporation with the pEF1a-CRE-T2A-mCh plasmid as described above. Single colonies were picked after 6 days, and cassette excision was assessed via PCR with the following primer combinations:

LA

**AP36**: 5′-GACACCTACTCAGACAATGC-3′ and

**AP68**: 5′-CAGGTCCTTAGTGACAGTCT-3′

LA′

**AP68** and

**JA20**: 5′-CGTCACCGCATGTTAGAAGA-3′

RA

**JA18**: 5′-AGTCAATCCGCCGAGTTGG-3′ and

**JA19**: 5′-GCTCAGATGGAGCAGTGGATG-3′

Heterozygous allele integrity of individual subclones was re-assessed by WT *CLYBL* locus PCR amplification with

**JA57**: 5′-TGCTTCAGCCCAGTTTCCAC-3′ and

**JA58**: 5′-TCACTTGAGCCCAGGACTTC-3′,

followed by PCR product purification and Sanger sequencing with JA21: 5′-TGAGGATTGAGCTCTCTTACCC-3′ and AP59: 5′-ACAAATCAAACATGGCTCAG-3′. This was complemented by confirming the presence of SNPs in the genome of KOLF2.1J at the *CLYBL* locus versus the human reference genome (GRCh38) upstream and downstream of the gRNA target site by PCR (upstream JA62: 5′-AAAGGGCAGTTTGCCTTGTC-3′ and JA63: 5′-AACAACACATGGTGCCATCC-3′; downstream JA66: 5′-GCTGCCAGGATCTTAAAGTC-3′ and JA67: 5′-CATTTACAGGGCACCTTCTC-3′) followed by Sanger sequencing (upstream JA64: 5′-GGCACTGAGGAGTGTCTAGG-3′ and JA65: 5′-CTCCTTCCGCTCCTCTAATC-3′; downstream JA68: 5′-CTCAGCCTCTCCAGCCTTAC-3′ and JA69: 5′-AGCCTGCCTCTCCTCTTCTG-3′). Pluripotency assessment by IF, off-target analysis, and karyotyping were carried out as described above. Mycoplasma testing was performed using the MycoAlert Plus detection kit (11680271, Lonza). The generated line was then utilised to facilitate differentiation into cortical patterned neurons^64^. Prior to neuronal differentiation, iPSCs were cultured in E8 Flex media. Neuronal differentiation was carried out by 3 days of exposure to doxycyline according to established protocols, and from DIV3 neurons were then matured in Neurobasal maintenance media supplemented with B27, BDNF, NT3 and Laminin up to DIV21^59,64^.

For PCR screens gDNA was extracted from single clones using DNeasy Blood & Tissue Kit (Qiagen, 69504). All general PCR screenings were carried out with KAPA Taq EXtra HotStart ReadyMix (Roche), and PCRs to amplify samples for Sanger sequencing were carried out using Platinum SuperFi II DNA Polymerase-High Fidelity (Thermo Fisher).

For pluripotency and differentiation assessment, cells were fixed and incubated with different antibodies and representative fluorescence images were captured using either an ECHO Revolve (BICO) or Opera Phenix (Revvity) microscope at 10× or 20× magnification. Differentiation capacity of iPSCs was tested using the induction protocol described, brightfield and red filter images were captured over the differentiation protocol to assess neuronal morphology and absence of excised mCherry signal.

### GFP-DPR_100_ transfection

All GFP-DPR_100_ transfections were performed in rodent primary cortical neuron cultures at 6 DIV. Cells were transfected with 0.5 µg DNA per well for either EGFP alone, GFP-PR_100_, GFP-GR_100_ or GFP-GA_100_ using 1 µl per well of Lipofectamine-3000 (Invitrogen) in OptiMEM media (Gibco), according to the manufacturer protocol. After 4 h, transfection media was replaced with the original culture media. At 72 h post-transfection, cells were fixed for immunofluorescence staining.

### siRNA transfection

Control and Syntaxin17 siRNA stocks were resuspended in RNase-free water to create 100 µM stock solutions according to manufacturer instructions (Accell). At DIV5, rodent primary cortical neurons were treated with 1 µM siRNA diluted in normal culture media.

### Retroviral infection of fibroblasts

To generate a stable mCherry-GFP-LaminB1^S391A^ cell line, 10 µg pBabe-mCherry-GFP-LaminB1^S391A^ was transfected into Phoenix retroviral packaging cells (ATCC, CRL-3213). Media was replenished the following day, collected 48 h later and passed through a 0.45 µm filter. Filtered media was then added to MRC-5 fibroblasts with 8 µg/ml polybrene (Santa Cruz, sc-134220). Media was replaced the following day, with 1 µg/ml puromycin added for selection after 48 h until all non-transduced control cells were dead.

### In vitro immunofluorescence staining

All cells were fixed with 4% paraformaldehyde for 10 min at room temperature and permeabilised with 0.1% Triton ×-100 (Sigma). Non-specific immunoreactivity was blocked with 1% BSA or 5% NGS for 30 min at room temperature. Primary antibody incubation was performed for 1 h at room temperature.

Primary antibodies: rabbit anti-LaminB1 1:500 (Abcam, ab16048); mouse anti-LaminB1 1:500 (Proteintech, 66095-1-IG); mouse anti-Mab414 1:500 (Abcam, ab24609); guinea-pig anti-p62 1:500 (Progen, GP62-C); rabbit anti-cleaved PARP-1 1:500 (Abcam, ab32561); rabbit anti-cleaved caspase-3 1:400 (Cell Signalling Technology, 9661S); chicken anti-MAP2 1:1000 (Abcam, ab5392); rabbit anti-p21 1:500 (Cell Signalling Technology, 2947); mouse anti-phospho-γH2AX 1:1000 (Sigma, 05-636); mouse anti-SSEA-4 1:500 (sc-21704, Santa Cruz); rabbit anti-FUS 1:1000 (hpa008784, Sigma); mouse anti-TRA-1-81 1:50 (sc-21706, Santa Cruz); rabbit anti-NANOG 1:200 (ab21624, Abcam); goat anti-SOX2 1:500 (sc-17320, Santa Cruz); mouse anti-Ki67 1:600 (BD-550609, BD Biosciences); mouse anti-TRA-1-60 1:50 (sc-21705, Santa Cruz); rabbit anti-OCT4 1:500 (ab19857, Abcam).

Secondary antibody incubation was performed for 2 h at room temperature. Secondary antibodies, all 1:500: goat anti-rabbit Alexa Fluor 488 (Invitrogen, A11034); anti-mouse Alexa Fluor 546 (Invitrogen, A11030); anti-guinea pig Alexa Fluor 555 (Invitrogen, A21435); anti-rabbit Alexa Fluor 546 (Invitrogen, A11035); anti chicken Alexa Fluor 633 (Invitrogen, A21103); goat anti-mouse AlexaFluor488 (A11029); donkey anti-mouse AlexaFluor 488 (A21202); goat anti-mouse IgG1 AlexaFluor 633 (A21126); goat anti-rabbit AlexaFluor 488 (A11008); donkey anti-rabbit AlexaFluor 546 (A10040); donkey anti-goat AlexaFluor 546 (A11056); donkey anti-goat AlexaFluor 633 (A21082). Cells were incubated with 1 µg/ml Hoechst-33342 (Invitrogen, H3570) for 10 min at room temperature. Primary neurons on coverslips were then mounted on slides with Dako (Agilent, S3023), all other cell preparations were imaged directly in microplates.

### TUNEL assay

TUNEL assay was carried out in fixed, permeabilised cells or *Drosophila* whole brains with a commercial kit (Roche, 12156792910) according to manufacturer instructions. Prior to the TUNEL assay, positive control samples were incubated with DNase I (Promega, M6101) in 50 mM Tris-HCl (pH 7.5) and 1 mg/ml BSA at 37 °C.

### Lactate dehydrogenase (LDH) assay

LDH release levels was measured in media collected from SH-SY5Y using a commercial kit (CytoTox 96^®^ Non-Radioactive Cytotoxicity Assay, Promega, G1780) with a standard 96-well plate reader (CLARIOstar, BMG LABTECH).

### Senescence β-galactosidase staining

SA-β-gal staining was performed on Sh-SY5Y cells with a commercial kit (Cell Signalling, 9860) according to manufacturer instructions.

### BODIPY™ staining

BODIPY™ 581/591 C11 (ThermoFisher) was used as a sensor of lipid peroxidation. A 10 mM stock of this sensor was prepared in DMSO and added to the cells at 10 μM and incubated for 30 min at 37 °C prior to fixation.

### Extracellular Vesicle Isolation

Conditioned media was harvested from SH-SY5Y cells treated with BafilomycinA1 or DMSO for 24 h and centrifuged at 300 × *g* for 5 min to remove dead cells. The supernatant was then extracted into ultracentrifuge tubes (Beckman Coulter, 344058) for processing into distinct pellet fractions corresponding to apoptotic bodies (“8 K” fraction), larger extracellular vesicles (“20 K” fraction) or smaller extracellular vesicles (“100 K” fraction). To obtain these fractions, sequential ultracentrifugation was carried out at 4 °C (SW 32 Ti rotor, Optima XPN-80, Beckman Coulter). For each fraction a wash step was included whereby the supernatant from the first ultracentrifugation was removed and temporarily stored at 4 °C, whilst 1× PBS was added to the pellet and ultracentrifugation repeated at the same speed and duration. The ultracentrifugation steps were as follows. 8 K pellet fraction: 8000 × *g* for 30 min; 20 K pellet fraction: 20,000 × *g* for 60 min; 100 K fraction: 100,000 × *g* for 70 min. Once isolated all final clean pellets obtained were stored at −70 °C prior to further biochemical processing. Total protein and double-strand DNA measurements of the EV fractions were obtained by UV–Vis spectrophotometry. 2 μL of each sample was loaded onto the pedestal of a Thermo Scientific™ NanoDrop™ One Microvolume UV–Vis Spectrophotometer and blanked with 0.1 μM filtered PBS between samples. Measurements were taken in duplicate, and the average of those measurements are reported.

### Nuclear-cytoplasmic fractionation

To isolate nuclear proteins, cells were transferred from 60 mm dishes to 1.5 mL Eppendorfs by mechanical scraping with 500 μL of fractionation buffer. Cells were then incubated for 15 min on ice with occasional vortexing. After this, using a 1 mL syringe, the cell suspension was passed through a 27-gauge (G) needle for 10–15 times, and this was left on ice for another 20 min. Cell suspension was then centrifuged for 5 min at 3000 rpm. The supernatant, containing the cytoplasmic and membrane fraction was separated. The pellet was washed again in 500 μL of fractionation buffer and passed through a 25 G needle 10–15 times. The suspension was centrifuged at 3000 rpm for 10 min. Supernatant was discarded and pellet containing the nuclei was resuspended in PK lysis buffer. Finally, nuclear fraction was sonicated for 5 s on ice (power setting: 2- continuous) to shear genomic DNA and homogenise the lysate.

### Immunoprecipitation

Before starting, protein G Dynabeads™ were resuspended by vortexing and 50 μL were transferred into a clean 1.5 mL Eppendorf. Alongside, the rabbit anti-LaminB1 (Abcam, ab229025) antibody was diluted 1:100 in PK lysis buffer. Using a magnetic separation rack, the solution containing the Dynabeads was separated, and the antibody was added into the beads and incubated for pre-loading for 1 h at 4 °C. Thereafter, the supernatant was removed and replaced by 200 μL of PK lysis buffer, followed by 100 μL of protein sample and incubated for 4 h at 4 °C on a roller for immunoprecipitation. After incubation, the beads were washed 3 times with 200 μL of PK lysis buffer. For protein elution, supernatant was removed and replaced by 40 μL of 2× Laemli buffer (10% β-mercaptoethanol), the mix was resuspended and boiled for 10 min.

### Western blots

Whole cell lysates or snap frozen *Drosophila* heads were prepared for Western blotting as follows. Cells were trypsinised, resuspended in RIPA buffer and incubated on ice for 30 min. In the cases where it was necessary, RIPA buffer was supplemented with phosphatase inhibitors (Milipore). *Drosophila* were mechanically lysed in Laemmli buffer. Samples were centrifuged at 13,000 rpm in a standard benchtop centrifuge at 4 °C for 5 min. The supernatant was then extracted into fresh tubes. Pierce BCA protein assay was performed to determine total protein concentration of samples, using a commercially available kit (ThermoFisher Scientific, 23225) according to instructions.

Samples were boiled in 1× Laemmli buffer with 10% β-mercaptoethanol at 95 °C for 5 min. Separation was then carried out by SDS-PAGE by loading 30 µg of protein, and then transferred onto nitrocellulose membrane (ECL, 0.2 µm pore size, Amersham GE) using a Mini-PROTEAN Tetra Cell tank (BioRad). Non-specific binding was blocked with 5% (w/v) milk powder or BSA in TBST overnight at 4 °C. Primary antibodies were incubated overnight at 4 °C. Primary antibodies: rabbit anti-GAPDH 1:2000 (Cell Signalling Technology, 2118); rabbit anti-LaminB1 1:1000 (Abcam, ab16048); rabbit anti-β-actin 1:1000 (Signalway, 21338); mouse anti-p62 1:1000 (Abnova, H00008878-1701); rabbit anti-p62 1:1000 (Cell Signaluing, 5114); mouse anti-p16 1:1000 (Santa Cruz, sc-56330); mouse anti-IL8 1:1000 (Abcam,ab18672); rabbit anti-IL1α 1:1000 (Abcam, ab9614); mouse anti-β-tubulin 1:1000 (DSHB, E7), mouse anti-*Drosophila­* LaminB 1:1000 (DHSB, ADL67.10), mouse anti-p53 1:2000 (Santa Cruz, sc-126), mouse anti-phospho-γH2AX 1:1000 (Sigma, 05-636), rabbit anti-p21 1:1000 (Cell Signalling Technology, 2947). Anti- HRP- or dye-conjugated secondary antibodies in 5% (w/v) milk powder or BSA in TBST were incubated for 1 h at room temperature. Secondary antibodies: goat anti-rabbit IgG (CalbioChem, DC03L); goat anti-mouse IgG (CalbioChem, 3239294101); goat anti-mouse IRDye 800 Cw 1:10,000 (LI-COR, D10825-15); goat anti-rabbit IRDye 680 RD (LI-COR, D11102-15), all 1:10,000. Specific protein signals were detected by chemiluminescence using SuperSignal West Pico Chemiluminescent Substrate (ThermoFisher Scientific, 34579) or via dye fluorescence using an Odyssey Imaging System (LI-COR).

To prepare isolated extracellular vesicle samples for Western blot, pellets were resuspended in 1× PBS and diluted 1:3 in RIPA buffer. Total protein concentration of samples was determined using a commercial micro-BCA kit (ThermoFisher Scientific, 23235) according to instructions. 2× Laemmli buffer with 10% β-mercaptoethanol was then added 1:1 to the pellet sample volume in the ultracentrifuge tube. Tubes were then placed in boiling water for 5 min.

### LaminB1 ELISA

Quantitative measurement of LaminB1 protein concentration in extracellular vesicle pellet samples was carried out by ELISA using a commercially available kit (Abcam, ab252351-10001) according to instructions.

### NanoParticle tracking analysis

Extracellular vesicle samples in 0.1 μM double-filtered 1× PBS were loaded onto a Nanosight NS300 with 488 nm scatter laser and high sensitivity camera (Malvern Instruments Ltd., Malvern, UK). Nanoparticle tracking and quantification of size and number of imaged particles was performed with NTA2.1 software (Nanosight, Malvern, UK).

### p38 and LaminB1 in vitro kinase assay

Expression and purification of recombinant active p38α and a LaminB1^31–385^ fragment was performed as previously described in ref. ^65^. An in vitro kinase assay was performed whereby 1 mg/ml LaminB1_313–586_ was incubated with either 30 or 60 ng active p38α for either 0.5, 1 or 2 h. The reaction was carried out in 1× kinase buffer (25 mM Tris/HCl pH 7.5, 5 mM β-glycerolphosphate, 2 mM DTT, 0.1 mM Na_3_VO_4_, and 1 mM MgCl2). ATP (550 μM) was added to the incubation mixture to start the reaction at 37 °C. At the end of reaction, the samples were collected with 2× sample buffer (20% glycerol, 6% SDS in 0.12 M Tris pH 6.8, 10% β-mercaptoethanol, and 0.4% Bromophenol blue), heated to 95 °C for 10 min, and run on 10% SDS gel under denaturing conditions. Western blot was then executed as previously outlined, staining with Pro-Q Diamond Phosphoprotein Gel Stain (ThermoFisher Scientific, P33300).

### *Drosophila* experiments

All fly stocks were routinely maintained at 18 °C in an incubator with a standard 12-h light cycle, on a standard fly food mixture of yeast, agar, and cornmeal with nipagin and propionic acid. For ageing experiments desired progeny were maintained at 29 °C and 60% humidity with a 12-h light cycle.

The following *Drosophila* stocks were used: *Elav-Gal4*, *UbiGal80ts, Stx17*^*LL*^*, UAS-PR*_*8*_*, UAS-PR*_*64*_*, UAS-GR*_*8*_*, UAS-GR*_*64*_*, UAS-GA*_*8*_*, UAS-GA*_*64*_*, UAS-Dronc*^*DN*^*[C318G], UAS-p38α* RNAi (VDRC stocks: 34238GD, 102484KK, 52277GD), *UAS-*p38β RNAi (VDRC stock: 108099KK), *w*^*1118*^*; LamB*^*T413A*^ *{PBacDsRed}/CyO*, *w*^*1118*^*; LamB*^*T413A*^*/CyO, w*^*1118*^ (generated by Well Genetics).

All non-G_4_C_2_ *UAS-DPR*_8/64_ stocks with a 3′ HA tag are previously described in ref. ^47^. To obtain flies pan-neuronally expressing inducible *UAS-DPR*_*8/64*_ constructs, *Elav-Gal4;UbiGal80ts* virgins were crossed to *UAS-DPR*_*8/64*_ males and maintained at 18 °C. F1 progeny were transferred to 29 °C for ageing for all experimental procedures. To obtain adult flies pan-neuronally co-expressing inducible *UAS-PR*_*8/64*_ constructs and *UAS-p38 RNAi*, *UAS-PR*_*8/64*_ and *UAS-p38* RNAi virgins were separately crossed to appropriate balancer chromosome stocks. Balanced virgins from F1 progeny, which incorporated *UAS-p38* RNAi were then crossed to balanced F1 males incorporating the *UAS-PR*_*8/64*_. Finally, F1 generation males from this cross, incorporating both *UAS* constructs, were crossed to *Elav-Gal4;UbiGal80ts* virgins.

Lifespan analysis was performed as previously described in ref. ^66^. Data are presented as survival curves and statistical comparisons between 8 and 64 repeat length genotype pairs for each DPR were carried out by log-rank test.

Negative geotaxis assays were performed as previously described^66^. Number of flies climbing to each cm increment was scored, and a genotype average was calculated for each timepoint across 5 trials. Flies which jumped or did not carry out a vertical climb in one movement burst were excluded for that trial.

Adult fly brain immunofluorescence was performed as previously described in ref. ^66^. For all immunofluorescence results shown, adult female fly brains were dissected at 15 days of ageing at 29 °C just prior to end-stage of lifespan of the most toxic DPR genotypes. For results where *w*^*1118*^ L3 stage larvae brains are shown as positive apoptosis controls, the same process was applied.

Primary and secondary antibody incubations were carried out overnight at 4 °C. Primary antibodies: anti-*Drosophila* LaminB 1:500 (DSHB, ADL67.10); anti-HA tag 1:100 (Sigma, H6908); anti-cleaved-Dcp-1 1:200 (Cell Signalling Technology, 9578); anti-cleaved caspase-3 1:200 (Cell Signalling Technology, 9661S); anti-Ref(2)P 1:500^67^. Secondary antibodies: 1:200 goat anti-mouse AlexaFluor 488 (Invitrogen, A11001) and goat anti-rabbit AlexaFluor 546 (Invitrogen, A11035). 1 µg/ml Hoechst-33342 nuclear stain was applied for 10 min at room temperature. Brains were mounted on slides with Dako (Agilent, S3023).

### Human *Post-Mortem* tissue immunohistochemistry

Human *post-mortem* paraffin-embedded 7 µm thickness frontal cortex sections were obtained from the London Neurodegenerative Diseases Brain Bank under Health Research Authority REC reference 18/WA/0206. Clinical and neuropathological case details are described in Supplementary Data 3.

All immunofluorescence staining, image acquisition and analysis of sections was carried out blind. Sections were warmed and dewaxed via short sequential washes in: xylene twice, 95% IMS twice and 95% IMS, followed by rinsing in distilled water. Antigen retrieval was carried out by boiling sections in pH 9.0 Tris-EDTA buffer (10 mM Tris base, 1 mM EDTA solution, 0.05% Tween 20), once cooled sections were briefly rinsed in PBS. Blocking of non-specific antibody binding was carried out by incubating sections in a blocking solution of 5% NGS, 1% BSA and 0.5% Triton ×-100 in PBS for 1 h. Sections were incubated with primary antibodies overnight at 4 °C. Primary antibodies: chicken anti-MAP2 1:200 (Abcam, ab5392), rabbit anti-LaminB1 1:500 (Abcam, ab16048). Secondary antibody incubation was carried out for 1 h. Secondary antibodies: goat anti-rabbit AlexaFluor 488 (Invitrogen, A11034) and goat anti-chicken AlexaFluor 633 (Invitrogen, A21103) both 1:250. Autofluorescence was quenched with Sudan Black B (199664, Sigma) solution for 10 min, followed by wash steps in 70% ethanol, distilled water, and PBS. Sections were incubated with 1 µg/ml Hoechst-33342 for 10 min.

### K-means cluster analysis

Clustering analysis of single-cell data from LaminB1 and MAP2 immunofluorescence parameters measured in human *post-mortem* frontal cortex was executed in *R* using the *kmeans* function from *stats* package (version 4.1.1), with parameters *k* = 4, *iter.max* = 100 and *nstar*t = 10. Input variables used were: LaminB1 nuclear circularity, cytoplasm area, nuclear area, or all of the above plus LaminB1 cytoplasm puncta (measured per area of cytoplasm and normalised for post mortem delay). The most appropriate number of clusters *k* was pre-determined via the within-cluster sum of squares method, whereby within-cluster sum of squares is plotted against increasing iterations of *k* to generate an elbow plot.

Within each identified cluster, statistical comparison of frequency of cells from different neurodegenerative disease or control categories was carried out in *R* by *χ*^2^ test followed by post-hoc pairwise binomial test with Bonferroni correction, implemented by *chisq.test* and *binom.test* functions respectively within the *stats* package.

### Atomic force microscopy

AFM was carried out on live PtK2 cells treated either with DMSO or 10 nM Bafilomycin for 24 h. All AFM experiments were executed on a BioScope Resolve system (Bruker) mounted on an AxioObserver.Z1 optical microscope (Zeiss) with 37 °C temperature control. Prior to AFM assay, average z-depth location of the nuclear surface and the z-depth of cytoplasm above the nucleus were determined by confocal microscopy visualisation of a sample of PtK2 cells where cytoskeleton and nucleus had been stained by immunofluorescence for actin and DAPI, respectively. Average distance from the cell surface to nuclear surface was found to be approximately 1 µm. From this, cytoplasmic indentation during experiments was estimated to take place for probe depth distance 0–1 µm (where 0 = point of contact), as cytoplasm is much softer than the nucleus and is therefore compressed first. Nuclear surface indentation was estimated to take place from 1 µm to maximal indentation depth (<2 µm). This was confirmed by a significant upward change in the force-indentation plot starting at 1 µm indentation.

Colloidal probes (PT.Si02.SN2.5, Novascan) were calibrated using the thermal noise method and then used for experimental AFM measurements in force curve mode above individual nuclei whose locations were determined by bright field microscopy. The following probe parameters were used: 8 µm ramp, 16 µm/s tip velocity, 15 nN force threshold. This was repeated for 200 cells for each treatment condition, for 3 different cell cultures.

Using a bespoke *Python* program^68^, elasticity was computed by fitting force indentation data in two different indentation ranges corresponding to cytoplasmic and nuclear elasticities.

### Confocal microscopy

Image acquisition of immunofluorescence for all SH-SY5Y experiments was carried out directly in 96-well microplates using an Opera Phenix high-content imaging system (PerkinElmer). Confocal scanning was carried out using a 60 × 1.15 NA water immersion objective. Maximum intensity projection images were compiled from z-stacks.

All other fluorescence image acquisition was carried out using an A1R inverted confocal microscope (Nikon), with either a 20 × 0.75 NA air objective or a 60 × 1.49 NA oil immersion objective. For in vitro immunofluorescence, maximum intensity projections were generated from z-stacks of 0.125 µm depth planes, of sufficient total to capture the entire z-height of the cells imaged.

For *Drosophila* whole brain, up to 20 single-plane images were obtained at 5 µm z-plane increments in large image mode stitching nine 1024-pixel tiles together.

For human *post-mortem* frontal cortex sections, images were acquired in a single z-plane in a series of 4 consecutive imaging locations starting from the tissue edge and working in towards the boundary between grey and white matter, determined from MAP2 staining morphology. Two 4-image sequences were obtained per case, from 2 independent starting points, from 1 single tissue section. Each image was comprised of nine 1024 × 1024-pixel tiles stitched together in large image field mode.

### Immunofluorescence quantification

In all instances, regularity of LaminB1 nuclear morphology was determined using circularity, defined as: circularity = perimeter^2^/4π × area. Using *Python* (v 3.8.13), all quantification of LaminB1 nuclear circularity in vitro was carried out using the *nuclei* model of the Cellpose algorithm^69^ to segment nuclei masks based on LaminB1 immunofluorescence. Circularity of masks was then calculated using the *Skimage* package.

Analysis of all SH-SY5Y immunofluorescence was carried out using Harmony (v 4.9, PerkinElmer). A basic pipeline was developed to identify and segment cells utilising “Find Nuclei” and “Find Cytoplasm” functions with appropriate filter parameters to capture the cell population. The “Calculate Intensity” function was used to quantify fluorescence intensity. The “Find Spots” function was used to quantify cytoplasmic p62 or LaminB1 puncta, or nuclear γH2ax foci within appropriate respective ROIs.

Analysis of primary neuron and human *post-mortem* immunofluorescence was carried out in NIS Elements Advanced Research Software (v5.01.00, Nikon). For human *post-mortem* tissue, neuronal cytoplasm and nuclear ROIs were drawn manually on the basis of MAP2 and LaminB1 signal, respectively. Identified neurons were excluded if the LaminB1 nuclear ring cross-section was incomplete and incompatible with reliable circularity quantification. A fluorescence intensity threshold was applied to LaminB1 fluorescence to detect cytoplasmic puncta. Cytoplasm area was calculated by subtracting the nuclear ROI area from the MAP2 soma ROI area.

For quantification of cleaved caspase-3 immunofluorescence intensity in primary cortical neurons, nuclei were identified based on Hoechst intensity with object size and shape restrictions applied. A GFP-positive sub-population was identified based on GFP fluorescence intensity thresholding. Finally, GFP-positive neurons were identified based on MAP2 fluorescence intensity thresholding. In the case of GFP-GA_100_, GFP-intense spots were identified using GFP fluorescence intensity thresholding, and then cells were screened and selected manually based on whether an identified GFP-positive spot localised to a MAP2-positive soma area. For quantification of LaminB1 karyoptosis parameters in higher magnification images of single GFP-positive cells identified & captured during acquisition, cytoplasm ROIs were manually drawn based on MAP2 signal. Intensity thresholding was applied to LaminB1 immunofluorescence to generate a nuclear ROI for circularity measurement. A second intensity threshold with size and shape filters, was applied to LaminB1 immunofluorescence in the cytoplasm ROI to quantify puncta.

### Statistical analysis

Statistical tests utilised are described in figure legends and were carried out in GraphPad Prism (version 9.4.1) or RStudio (version 4.1.1). In the latter case, all tests were carried out using their eponymous function within the *stats* package, except for Dunnett’s post-hoc test, which was executed using the *DunnTest* function within the *DescTools* package (version 0.99.44). Where required to ensure equivalence of sample size, random sampling of datasets was carried out using the *sample* function within the *stats* package.

### Reporting summary

Further information on research design is available in the Nature Portfolio Reporting Summary linked to this article.

## Supplementary information

Supplementary Information

Description of Additional Supplementary Files

Supplementary Data 1

Supplementary Data 2

Supplementary Data 3

Reporting Summary

Transparent Peer Review file

## Source data

Source Data

## Data Availability

All proteomics data are available via ProteomeXchange with identifier PXD074564. Source data for other experiments are either provided with this paper in Supplementary files or deposited on Zenodo (see figure legends for details). Additional data, materials and details are available upon request. Source data are provided with this paper.
