# Supplementary material for: Karyoptosis mediates cell death and neurodegeneration upon proteotoxic stress
Source: Nat Commun. 2026 Jun 25;17:5135. doi: 10.1038/s41467-026-73802-w (PMC13303863; doi:10.1038/s41467-026-73802-w)
Supplement: Supplementary file 1 — Supplementary Information [file 41467_2026_73802_MOESM1_ESM.pdf]

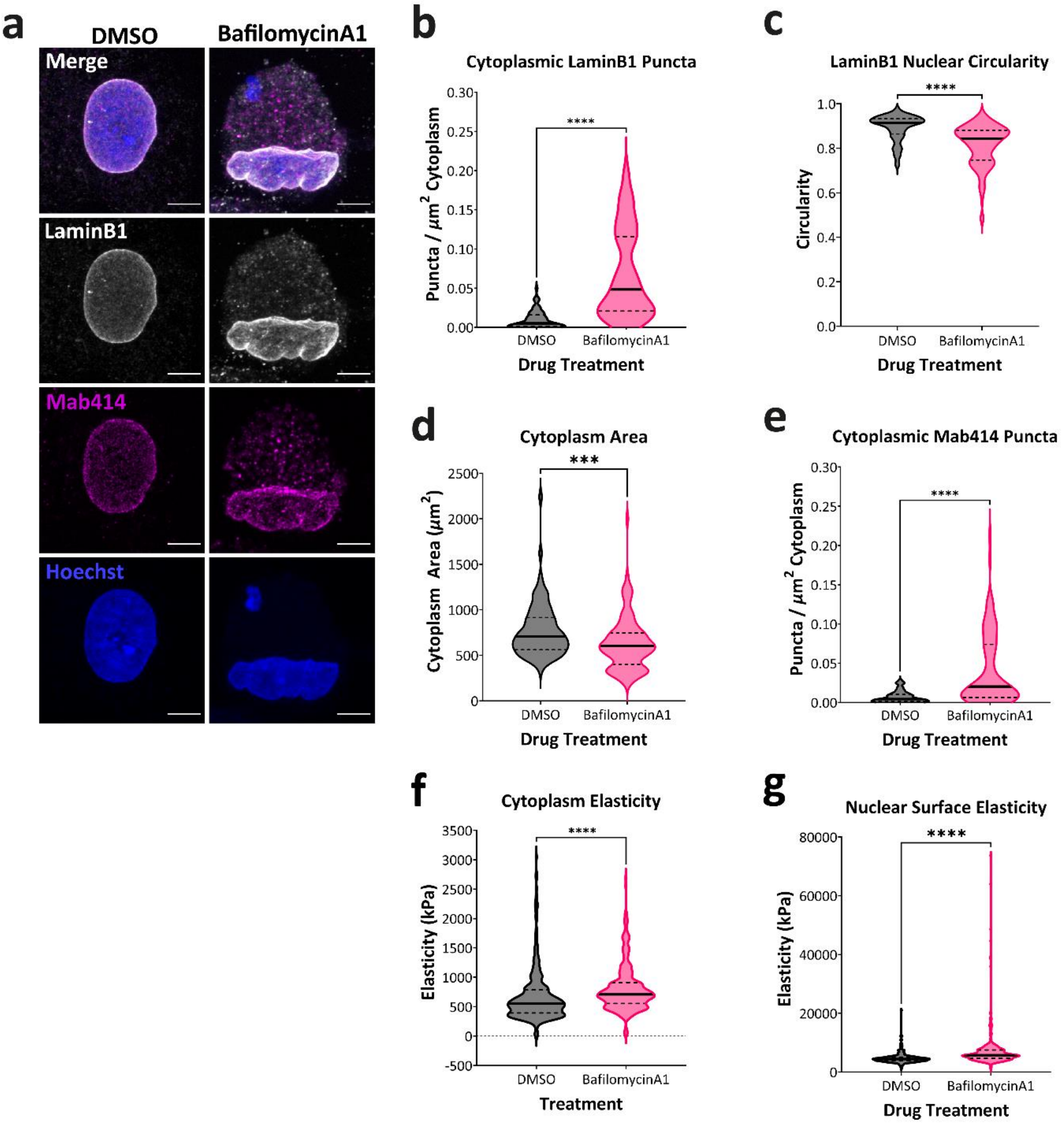

Supplementary Figure 1 - Pharmacological impairment of autophagic clearance triggers loss of nuclear integrity in PtK2 cells

a) Images of LaminB1, nuclear pore complexes (Mab414) and Hoechst immunofluorescence in cells treated with BafilomycinA1. Scale bars: 5μm. Quantification of cytoplasmic LaminB1 puncta (**b**), LaminB1 nuclear circularity (**c**), cytoplasm area (**d**) and cytoplasmic nuclear pore complexes (**e**). Cytoplasmic elasticity (**f**) and nuclear surface elasticity (**g**) measured by atomic force microscopy. **** = $p < 0.0001$, *** = $p < 0.001$. In violin plots (**b-g**) solid lines represent median, dashed lines represent 25th and 75th percentiles. Statistical analyses:

two-tailed unpaired t test (**b, c, d, e**) or two-tailed Mann-Whitney test (**f, g**). N (cells) = **b, d, e**: DMSO = 82, BafA1 = 104; **c**: DMSO =107, BafA1 = 118; **f & g:** DMSO = 300, BafA1 = 396.

$t = 9.279$, $p < 0.0001$ (**b**); $t= 7.586$, $p <0.0001$ (**c**); $t = 3.371$, $p = 0.0009$ (**d**); $t = 6.765$, $p < 0.0001$ (**e**); $U = 35090$, $p <0.0001$ (**f**); $U = 30293$, $p < 0.0001$ (**g**).

Raw data and further statistical information provided in the Source Data File and at doi 10.5281/zenodo.19813607.

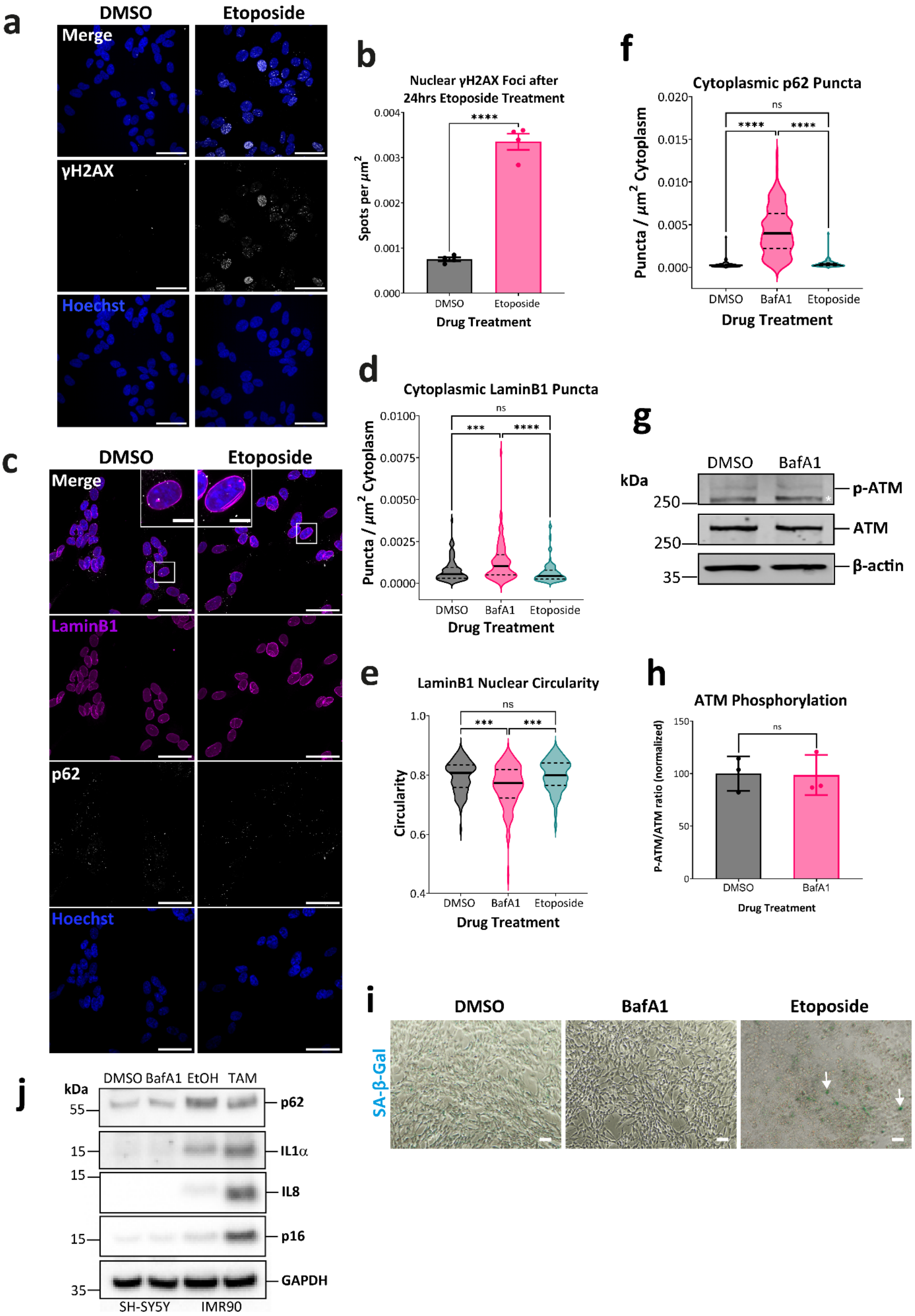
a
DMSO
Etoposide
Merge
γH2AX
Hoechst
b
Nuclear γH2AX Foci after 24hrs Etoposide Treatment
Spots per μm²
0.004
0.003
0.002
0.001
0.000
****
DMSO
Etoposide
Drug Treatment
f
Cytoplasmic p62 Puncta
Puncta / μm² Cytoplasm
0.020
0.015
0.010
0.005
0.000
ns
****
****
DMSO
BafA1
Etoposide
Drug Treatment
d
Cytoplasmic LaminB1 Puncta
Puncta / μm² Cytoplasm
0.0100
0.0075
0.0050
0.0025
0.0000
ns
***
****
DMSO
BafA1
Etoposide
Drug Treatment
g
DMSO
BafA1
kDa
250
250
35
p-ATM
ATM
β-actin
c
DMSO
Etoposide
Merge
LaminB1
p62
Hoechst
e
LaminB1 Nuclear Circularity
Circularity
1.0
0.8
0.6
0.4
ns
***
***
DMSO
BafA1
Etoposide
Drug Treatment
h
ATM Phosphorylation
P-ATM/ATM ratio (normalized)
150
100
50
0
ns
DMSO
BafA1
Drug Treatment
i
DMSO
BafA1
Etoposide
SA-β-Gal
j
kDa
DMSO
BafA1
EtOH
TAM
55
15
15
15
35
p62
IL1α
IL8
p16
GAPDH
SH-SY5Y
IMR90

Supplementary Figure 2 – Chemically induced DNA damage and senescence are insufficient to trigger nuclear lamina phenotypes observed in models of autophagic impairment

a) Immunofluorescence images of DNA damage marker γH2AX in SH-SY5Y cells treated with etoposide. **b)** Quantification of nuclear γH2AX foci. **c)** Immunofluorescence images of LaminB1, p62 and Hoechst in SH-SY5Y treated with etoposide, and quantification of LaminB1 cytoplasmic puncta (**d**), LaminB1 nuclear circularity (**e**) and cytoplasmic p62 puncta (**f**). **g)** Western blot for ATM and phospho-ATM from whole cell lysate of SH-SY5Y cells treated with BafA1. **h)** Quantification of p-ATM:ATM ratio. **i)** Examples of SA-β-Gal staining in SH-SY5Y cells treated with DMSO, BafA1 or etoposide. Only etoposide induces visible SA-β-Gal (white arrows). **j)** Western blot for SASP secreted cytokines IL8, IL1α and for p16 and p62 in SH-SY5Y cells treated with DMSO, BafA1 or etoposide.

Scale bars: 50µm (**a, c**), 10µm (**c**, inset). Error bars denote mean ± SEM (**b & k**). In violin plots solid lines represent median, dashed lines represent 25th and 75th percentiles (**d-f**). *** = p < 0.001, **** = p < 0.0001.

Statistical analyses: two-tailed unpaired t-test (**b & h**), one-way ANOVA and Tukey's post hoc multiple p=comparisons (**d-f**).

N (wells) = **b:** DMSO = 4, BafA1 = 4; (cells) = **d:** DMSO = 99, BafA1 = 100, Etoposide = 111, **e:** DMSO = 97, BafA1 = 108, Etoposide = 81; **f:** DMSO = 111, BafA1 = 149, Etoposide = 116.

t = 14.27, p < 0.0001 (**b**); F = 16.17, p < 0.0001 & Tukey's multiple comparisons DMSO vs BafA1 p = 0.0002, DMSO vs Etoposide p = 0.4142, Etoposide vs BafA1 p < 0.0001 (**d**); F = 11.31, p < 0.0001 & Tukey's multiple comparisons DMSO vs BafA1 p = 0.0004, DMSO vs Etoposide p = 0.8653, Etoposide v BafA1 p = 0.0001 (**e**); F=225.2, p < 0.0001 & Tukey's multiple comparisons DMSO vs BafA1 p <0.0001, DMSO vs Etoposide p =0.8959, Etoposide vs BafA1 p < 0.0001 (**f**).

Raw data and further statistical information provided in the Source Data File and at dois 10.5281/zenodo.19372262 and 10.5281/zenodo.18806148.

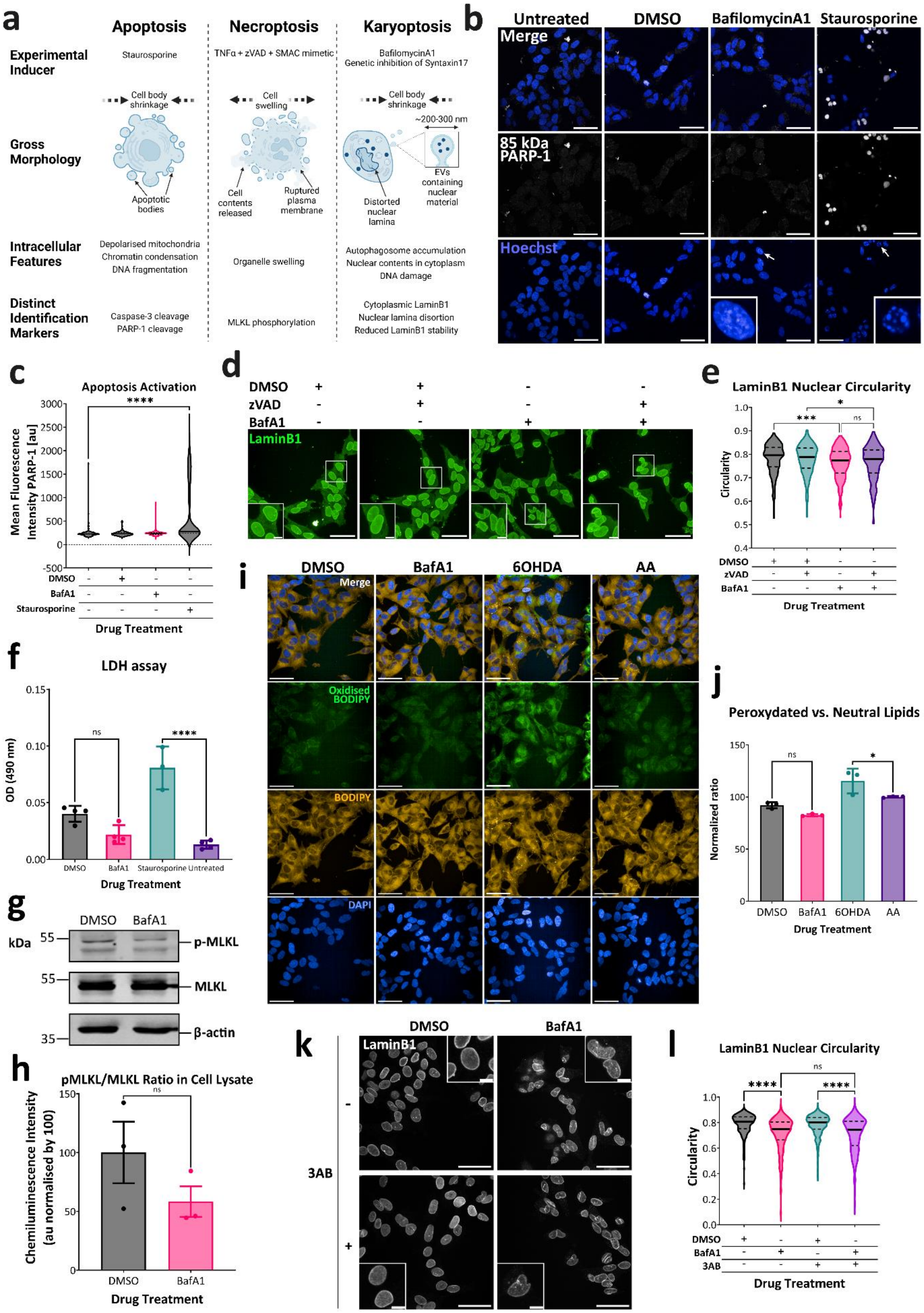

a
Apoptosis
Necroptosis
Karyoptosis
Experimental Inducer
Staurosporine
TNFα + zVAD + SMAC mimetic
BafilomycinA1
Genetic inhibition of Syntaxin17
Gross Morphology
Cell body shrinkage
Cell swelling
Cell body shrinkage
~200-300 nm
Apoptotic bodies
Cell contents released
Ruptured plasma membrane
Distorted nuclear lamina
EVs containing nuclear material
Intracellular Features
Depolarised mitochondria
Chromatin condensation
DNA fragmentation
Organelle swelling
Autophagosome accumulation
Nuclear contents in cytoplasm
DNA damage
Distinct Identification Markers
Caspase-3 cleavage
PARP-1 cleavage
MLKL phosphorylation
Cytoplasmic LaminB1
Nuclear lamina disortion
Reduced LaminB1 stability
b
Untreated
DMSO
BafilomycinA1
Staurosporine
Merge
85 kDa PARP-1
Hoechst
c
Apoptosis Activation
Mean Fluorescence Intensity PARP-1 [au]
DMSO
BafA1
Staurosporine
Drug Treatment
d
DMSO
zVAD
BafA1
LaminB1
e
LaminB1 Nuclear Circularity
Circularity
DMSO
zVAD
BafA1
Drug Treatment
f
LDH assay
OD (490 nm)
DMSO
BafA1
Staurosporine
Untreated
Drug Treatment
g
DMSO
BafA1
kDa
p-MLKL
MLKL
β-actin
h
pMLKL/MLKL Ratio in Cell Lysate
Chemiluminescence Intensity (au normalised by 100)
DMSO
BafA1
Drug Treatment
i
DMSO
BafA1
6OHDA
AA
Merge
Oxidised BODIPY
BODIPY
DAPI
j
Peroxydated vs. Neutral Lipids
Normalized ratio
DMSO
BafA1
6OHDA
AA
Drug Treatment
k
DMSO
BafA1
LaminB1
3AB
l
LaminB1 Nuclear Circularity
Circularity
DMSO
BafA1
3AB
Drug Treatment

Supplementary Figure 3 – Karyoptosis is distinct from other cell death mechanisms

a) Schematic highlighting key features distinguishing karyoptosis and other canonical cell death mechanisms. Created in BioRender. Mizielinska, S. (2026) https://BioRender.com/p96h033 **b)** Immunofluorescence images of cleaved 85 kDa apoptotic fragment of PARP-1 in SH-SY5Y cells treated with DMSO, BafA1 or apoptosis-inducer 1μM staurosporine for 24 hours. **c)** Quantification of nuclear 85 kDa PARP-1 immunofluorescence intensity. **d)** Images of LaminB1 immunofluorescence in SH-SY5Y cells treated with either DMSO or BafA1 ± the apoptotic caspase inhibitor z-VAD. **e)** Quantification of LaminB1 nuclear circularity upon caspase inhibition. **f)** LDH assay from SH-SY5Y cells untreated or treated with DMSO, BafA1 or 1μM staurosporine for 24 hours. **g)** Western blot probing phospho-MLKL, MLKL and loading control β-actin in whole cell lysate of SH-SY5Y treated with DMSO or BafA. **h)** Ratio quantification of p-MLKL (top band, corresponding to upward smear in MLKL) versus total MLKL. i) Fluorescence images of the lipid peroxidation sensor BODIPY 581/591 C11 in SH-SY5Y cells treated with either DMSO, BafA1, positive control 35μM 6-OHDA or vehicle control 0.02% ascorbic acid (AA). Oxidised lipids associated with ferroptosis are visualized in green, neutral lipids are visualized in orange. **j**) Quantification of peroxidated : neutral 89 lipid fluorescence intensity. **k)** Images of LaminB1 immunofluorescence in SH-SY5Y cells treated with either DMSO or BafA1± the PARP-1 and parthanatos inhibitor 3AB. **l)** Quantification of LaminB1 nuclear circularity upon caspase inhibition. Throughout this figure all uses of DMSO and BafA1 are 0.1% and 10nM concentration, respectively.

Scale bars (**b, d, i, j**): 50μm and (**j,** insets) 10μm. In violin plots solid lines represent median, dashed lines represent 25th and 75th percentiles (**c, e, l**). In bar plots error bars denote mean ± SEM (**f, h, j**). * = $p < 0.05$, *** = $p < 0.001$, **** = $p < 0.0001$.

Statistical analyses: Kruskal-Wallis test and Dunn's post-hoc multiple comparisons test (**c, e, l**), two-tailed unpaired t-test (**h**); one-way ANOVA and Tukey's post-hoc multiple comparisons test (**f, j**).

N (cells) = **c:** 200 per condition, **e:** DMSO = 334, DMSO + zVAD = 365, BafA1 = 344, BafA1 + zVAD = 318, **l:** DMSO = 299, BafA1 = 291, DMSO + 3AB = 346, BafA1 + 3AB = 266; (wells) **f:** DMSO, BafA1 & untreated = 4, staurosporine = 3, **j:** 3 per condition; (experiments) **h:** 3.

Kruskal-Wallis statistic = 151.7, p < 0.0001 & Dunn's multiple comparisons: untreated vs. DMSO p >0.9999, untreated vs. BafA1 p = 0.1697, untreated vs staurosporine p < 0.0001 (**c**); Krukal-Wallis statistic = 22.90, p <0.0001 & Dunn's multiple comparisons: DMSO + zVAD vs BafA1 + zVAD p = 0.0267, BafA1 vs BafA1 + zVAD p > 0.9999, DMSO vs BafA1 p = 0.0002 (**e**); Kruskal-Wallis statistic = 124.2, p <0.0001 & Dunn's multiple comparisons: DMSO vs BafA1 p < 0.0001, DMSO +3AB vs BafA1 + 3AB p <0.0001, BafA1 vs BafA1 + 3AB p > 0.9999 (**l**); F = 16.10, p < 0.0001, Tukey's multiple comparisons: DMSO vs BafA1 p = 0.2713, untreated vs staurosporine p < 0.0001 (**f**); F = 36.35, p < 0.0001, Tukey's multiple comparisons DMSO vs BafA1 p = 0.0015, 6-OHDA vs AA p = 0.0213 (**j**); t = 1.427, df = 4, p = 0.2268 (**h**).

Raw data and further statistical information provided in the Source Data File and at doi 10.5281/zenodo.18815977.

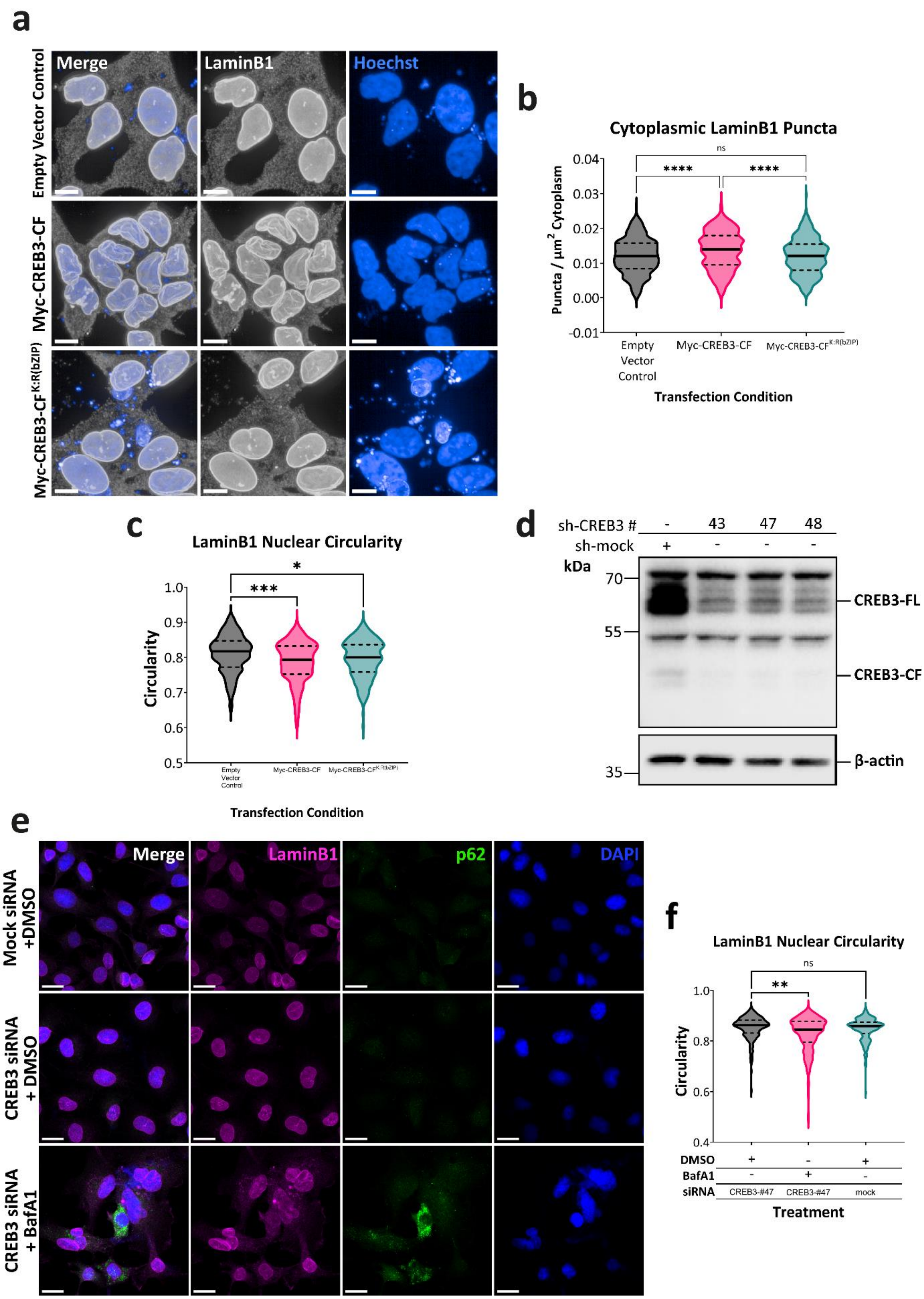
a
Empty Vector Control
Myc-CREB3-CF
Myc-CREB3-CF K:R(bZIP)
Merge
LaminB1
Hoechst
b
Cytoplasmic LaminB1 Puncta
ns
****
****
Puncta / µm2 Cytoplasm
0.04
0.03
0.02
0.01
0.00
-0.01
Empty Vector Control
Myc-CREB3-CF
Myc-CREB3-CF K:R(bZIP)
Transfection Condition
c
LaminB1 Nuclear Circularity
*
***
Circularity
1.0
0.9
0.8
0.7
0.6
0.5
Empty Vector Control
Myc-CREB3-CF
Myc-CREB3-CF K:R(bZIP)
Transfection Condition
d
sh-CREB3 #
-
43
47
48
sh-mock
+
-
-
-
kDa
70
55
35
CREB3-FL
CREB3-CF
β-actin
e
Mock siRNA +DMSO
CREB3 siRNA + DMSO
CREB3 siRNA + BafA1
Merge
LaminB1
p62
DAPI
f
LaminB1 Nuclear Circularity
ns
**
Circularity
1.0
0.8
0.6
0.4
DMSO
+
-
+
BafA1
-
+
-
siRNA
CREB3-#47
CREB3-#47
mock
Treatment

Supplementary Figure 4 - CREB3 does not mediate karyoptosis induced by proteotoxic stress

a) LaminB1 and Hoechst immunofluorescence images of SH-SY5Y cells transfected with either an empty vector control, Myc-CREB3-CF or mutated inactive Myc-CREB3-CFK:R(bZIP) and subsequent quantification of cytoplasmic LaminB1 puncta (**b**) and of LaminB1 circularity (**c**). **d**) Western blot for CREB3 from cell lysates of SH-SY5Y treated with either mock or three different CREB3 specific shRNA. All cells have been treated with the proteasome inhibitor MG132, 10µM for 4 hours, to stabilise the CREB3 protein. **e**) LaminB1, p62 and DAPI immunofluorescence images of SH-SY5Y treated with a mock shRNA or CREB3 shRNA ± 82 BafA1 treatment. **f**) Quantification of LaminB1 nuclear circularity. Scale bars: 10 µm (**a**), 20 µm (**e**).

In violin plots solid lines represent median, dashed lines represent 25th and 75th percentiles (**b, c, f**).

N = (cells) **b:** Empty Vector Control = 361, Myc-CREB3-CF = 499, Myc-CREB3-CFK:R(bZIP) = 499, **c:** Empty Vector Control = 195, Myc-CREB3-CF = 200, Myc-CREB3-CFK:R(bZIP) = 201, **f:** DMSO + CREB3 siRNA = 228, BafA1 + CREB3 siRNA = 167, DMSO + mock = 259.

Statistical analyses: one-way ANOVA and Tukey's post-hoc multiple comparisons test (**b**); Kruskal-Wallis test and Dunn's post-hoc multiple comparisons test (**c**, **f**). * $p < 0.05$, ** $p < 0.01$, **** $p < 0.0001$.

$F = 15.44$, ANOVA $p <0.0001$ and Tukey's multiple comparisons: Empty Vector Control vs Myc-CREB3-CF $p <0.0001$, Empty Control vs Myc-CREB3-CFK:R(bZIP) $p = 0.9918$, Myc-CREB3-CF vs Myc-CREB3-CFK:R(bZIP) $p <0.0001$ (**b**); Kruskal-Wallis statistic = 14.42, Kruskal-Wallis $p = 0.0007$ and Dunn's multiple comparisons: Empty Vector Control vs Myc-CREB3-CF $p = 0.0001$, Empty Vector Control vs Myc-CREB3-CFK:R(bZIP) $p = 0.0181$ (**c**); Kruskal-Wallis statistic = 12.05, Kruskal-Wallis $p = 0.0024$ and Dunn's multiple comparisons:

DMSO mock vs DMSO CREB3 siRNA p = 0.236, CREB3 siRNA + BafA1 vs CREB3 siRNA + DMSO p = 0.001 **(f)**.

Raw data and further statistical information provided in the Source Data File and at doi 10.5281/zenodo.18745789.

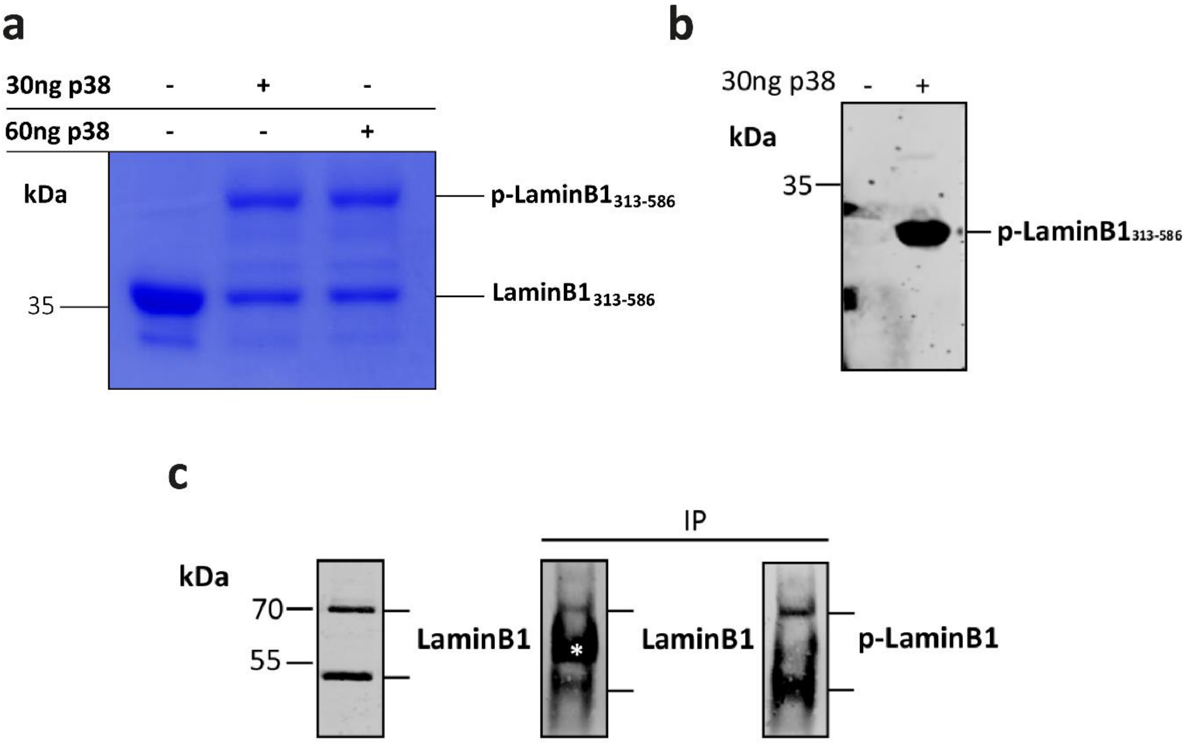

Supplementary Figure 5 – LaminB1 phosphorylation by p38 MAPK in karyoptosis

a) Coomassie blue stain for *in vitro* kinase assay with p38 (30ng or 60ng) and recombinant purified C-terminal (313-586) of LaminB1. **d**) *In vitro* kinase assay with 30ng p38, validating p-LaminB1 antibody utilised for immunoprecipitation. **e**) Western blots of LaminB1 from whole nuclear lysates of SH-SY5Y treated with BafA1 and of LaminB1 and phospho-LaminB1 with the newly raised antibody, after Immunoprecipitation of 126 LaminB1. Asterisk denotes the antibody high molecular chain band.

Raw data and further statistical information provided in the Source Data File and at doi 10.5281/zenodo.19911399.

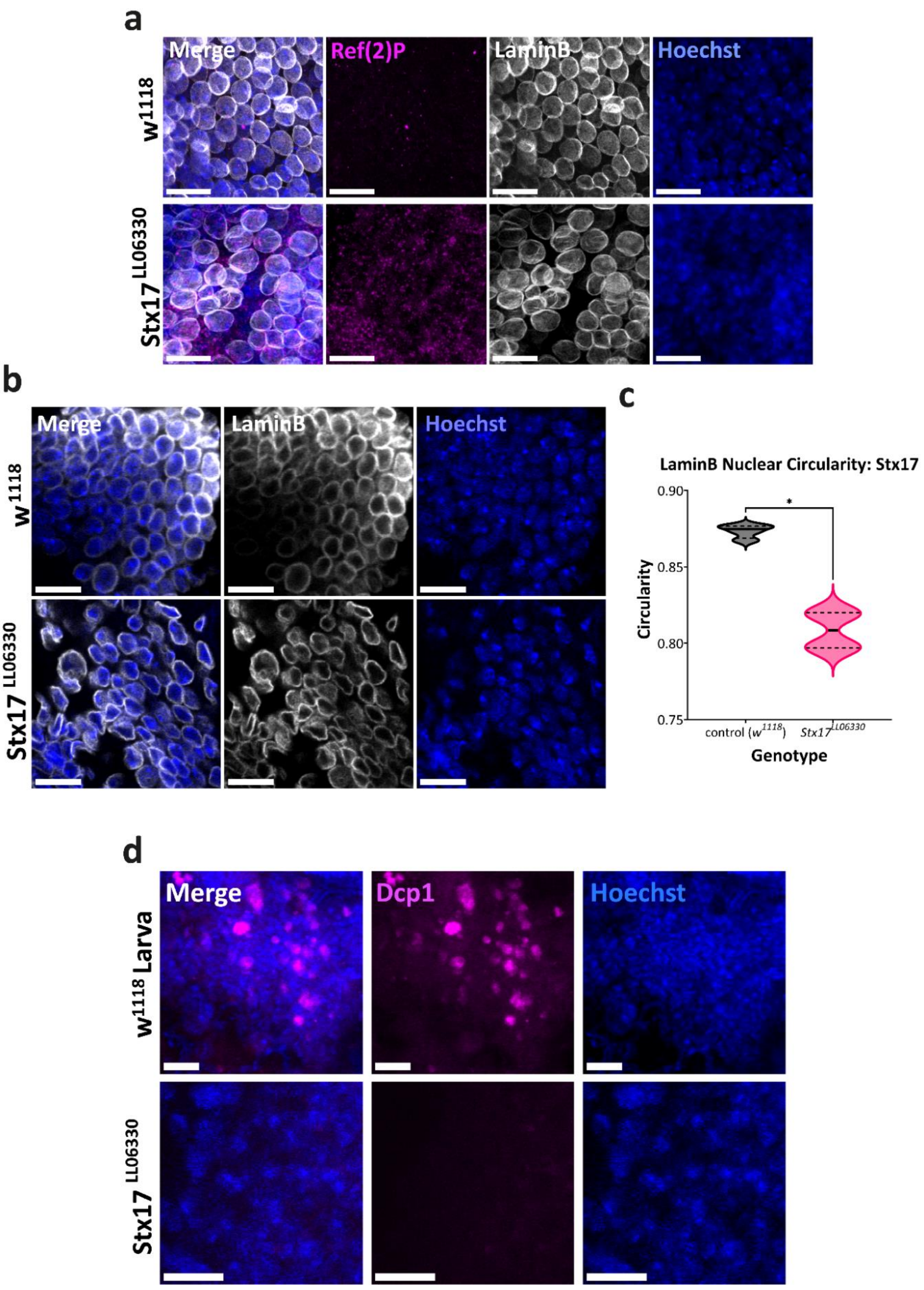
a
Merge
Ref(2)P
LaminB
Hoechst
w1118
Stx17 LL06330
b
Merge
LaminB
Hoechst
w1118
Stx17 LL06330
c
LaminB Nuclear Circularity: Stx17
0.90
0.85
0.80
0.75
Circularity
*
control (w1118)
Stx17LL06330
Genotype
d
Merge
Dcp1
Hoechst
w1118 Larva
Stx17 LL06330

Supplementary Figure 6 – Genetic model of autophagic clearance impairment in *Drosophila* recapitulates karyoptotis features

a) Immunofluorescence images of Drosophila p62 homologue, Ref(2)p in brain of adult *Syntaxin17*^{LL06330} flies. **b)** LaminB immunofluorescence images in adult brain of control (w^{1118}) or *Syntaxin17*^{LL06330} mutant *Drosophila*. **c)** Quantification of LaminB nuclear circularity. Violin plot solid lines represent median, dashed lines represent 25th and 75th percentiles. * = $p < 0.05$ **d)** Immunofluorescence images of *Drosophila* caspase-3 homologue, Dcp1 in wildtype (w^{1118}) L3 larvae undergoing developmental apoptosis, and brain of adults expressing *Syntaxin17*^{LL06330}. Scale bars: 5μm (**a, b**), 10μm (**d**).

Statistical analysis (**c**): two tailed Mann-Whitney test, p = 0.0286. N = 4 flies per group.

Raw data and further statistical information provided in the Source Data File and at doi 10.5281/zenodo.18838331.

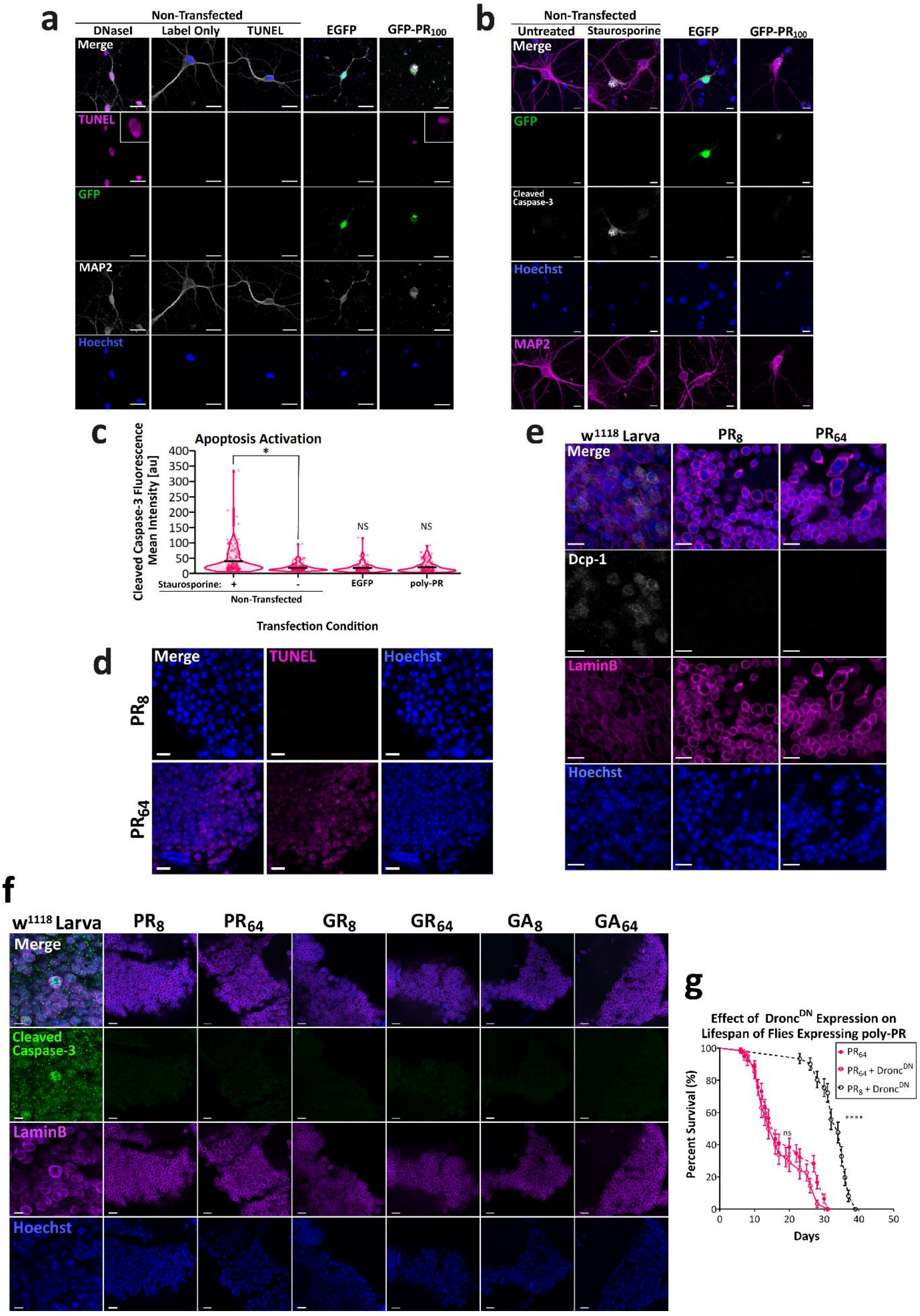
a
Non-Transfected
DNaseI
Label Only
TUNEL
EGFP
GFP-PR100
Merge
TUNEL
GFP
MAP2
Hoechst
b
Non-Transfected
Untreated
Staurosporine
EGFP
GFP-PR100
Merge
GFP
Cleaved Caspase-3
Hoechst
MAP2
c
Apoptosis Activation
Cleaved Caspase-3 Fluorescence Mean Intensity [au]
Staurosporine: + -
Non-Transfected
EGFP
poly-PR
NS
Transfection Condition
d
Merge
TUNEL
Hoechst
PR8
PR64
e
w1118 Larva
PR8
PR64
Merge
Dcp-1
LaminB
Hoechst
f
w1118 Larva
PR8
PR64
GR8
GR64
GA8
GA64
Merge
Cleaved Caspase-3
LaminB
Hoechst
g
Effect of DroncDN Expression on Lifespan of Flies Expressing poly-PR
PR64
PR64 + DroncDN
PR8 + DroncDN
Percent Survival (%)
Days
ns
****

Supplementary Figure 7 – Arginine-rich *C9ORF72* DPRs cause non-apoptotic cell death *in vitro* and *in vivo*

a) Rat primary cortical neurons expressing 100 repeat length GFP-tagged *C9ORF72* non-HRE DPRs combined with TUNEL assay fluorescence detecting cell death-associated DNA damage, and MAP2 immunofluorescence. Zoomed-in insets highlight representative single-cell examples of TUNEL positive nuclei. **b)** Images of cleaved caspase-3 immunofluorescence in rat primary cortical neurons expressing 100 repeat length GFP-tagged individual *C9ORF72* non-HRE DPRs. Non-transfected cells treated with 1µM staurosporine represent an apoptosis positive control. **c)** Quantification of cleaved caspase-3 immunofluorescence intensity in primary neurons. Horizontal error bar represents mean. **d)** Images of death-associated DNA damage detected by TUNEL assay fluorescence in *Drosophila* expressing either 8 or 64 repeat-length poly-PR. In all panels d-g DPRs and/or DroncDN expression is spatiotemporally driven in adult brain by the *Elav-Gal4;UbiGal80ts* driver. **e)** Dcp-1 and LaminB immunofluorescence in *Drosophila* brain expressing $PR_{8/64}$. Staining was carried out in w^{1118} larvae brain as an apoptosis positive control. **f)** Images of cleaved caspase-3, and LaminB immunofluorescence in the adult brain of *Drosophila* expressing HA-tagged 8 or 64 repeat length *C9ORF72* DPRs. Staining was carried out in w^{1118} larvae brain as an apoptosis positive control. **g)** Lifespan of flies expressing poly-$PR_8/_{64}$ ± a C318G dominant negative mutant Dronc caspase. Scale bars: 50 µm (**a**), 10 µm (**b**) 5 µm (**d-f**).

Error bars denote mean ± SEM (**g**).

Statistical analyses: one-way ANOVA and Dunnett's post-hoc test (**c**), Kaplan Meyer analysis with Gehan-Breslow-Wilcoxon test (**g**). * $p < 0.05$, **** $p < 0.0001$.

N = (cells, isolated from 2 rats) **c:** Non-transfected + staurosporine = 150, Non-transfected untreated = 150, EGFP = 93, poly-PR = 117; (flies) PR_{64} = 78, PR_{64} + DroncDN = 62, PR_8 + DroncDN = 61.

$F = 5.781$, $p = 0.0271$ and Dunnett's multiple comparisons test: non-transfected untreated vs non-transfected + staurosporine $p = 0.0437$, non-transfected untreated vs EGFP $p > 0.9999$, non-transfected untreated vs poly-PR $p = 0.9999$ (**c**); PR_8 + DroncDN vs PR_{64}: $\chi^2 = 96.45$, df = 1, $p < 0.0001$, PR_{64} + DroncDN vs PR_{64}: $\chi^2 = 2.76$, df = 1, $p = 0.2917$ (**g**).

Raw data and further statistical information provided in the Source Data File and at doi 10.5281/zenodo.19451986.

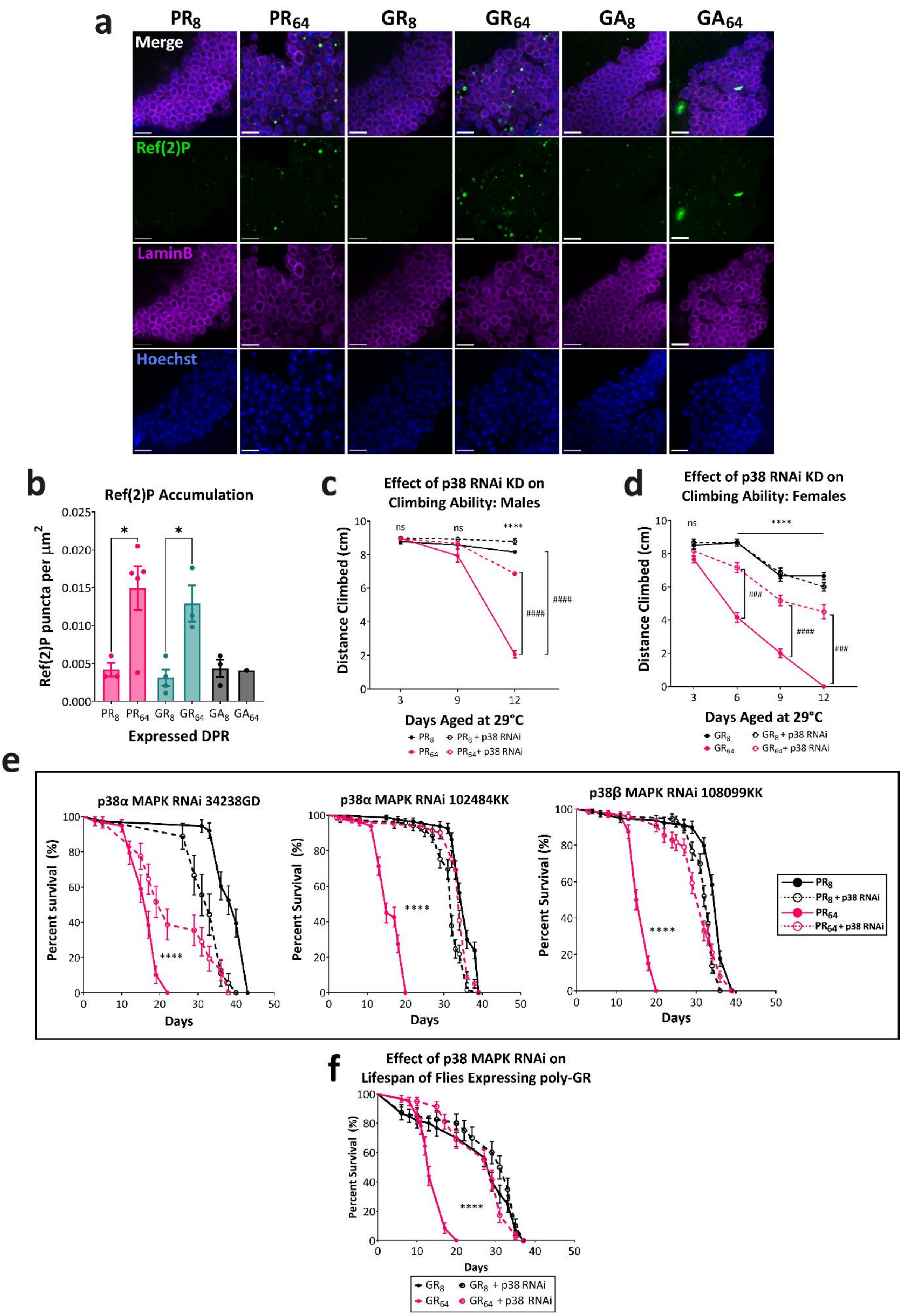
a
PR8 PR64 GR8 GR64 GA8 GA64
Merge
Ref(2)P
LaminB
Hoechst
b
Ref(2)P Accumulation
Ref(2)P puncta per µm2
Expressed DPR
c
Effect of p38 RNAi KD on Climbing Ability: Males
Distance Climbed (cm)
Days Aged at 29°C
PR8 PR8 + p38 RNAi
PR64 PR64+ p38 RNAi
d
Effect of p38 RNAi KD on Climbing Ability: Females
Distance Climbed (cm)
Days Aged at 29°C
GR8 GR8 + p38 RNAi
GR64 GR64+ p38 RNAi
e
p38α MAPK RNAi 34238GD
p38α MAPK RNAi 102484KK
p38β MAPK RNAi 108099KK
Percent survival (%)
Days
PR8
PR8 + p38 RNAi
PR64
PR64 + p38 RNAi
f
Effect of p38 MAPK RNAi on Lifespan of Flies Expressing poly-GR
Percent Survival (%)
Days
GR8 GR8 + p38 RNAi
GR64 GR64 + p38 RNAi

Supplementary Figure 8 – Arginine-rich C9ORF72 DPRs cause proteotoxic stress-linked neuronal karyoptosis and their effects are rescued by p38 MAPK downregulation

a) Ref(2)P and LaminB immunofluorescence in the adult brain of *Drosophila* expressing HA tagged 8 or 64 repeat length *C9ORF72* DPRs. In all panels DPRs and/or p38 RNAi expression is spatiotemporally driven in adult brain by the *Elav-Gal4;UbiGal80ts* driver. Scale bars: 5 µm. **b)** Quantification of Ref(2)P puncta accumulation. **c)** Climbing motor function of male flies expressing poly-$PR_{8}/_{64}$ ± p38α RNAi (52277GD). **d)** Climbing motor function analysis in female *Drosophila* co-expressing either GR_{8} or GR_{64} with p38 MAPK RNAi (52277GD) in the adult brain. **e**)

Lifespan of flies expressing poly-$PR_{8}/_{64}$ ± different p38α RNAi (left, 102484KK; middle, 34238GD) or ± p38β RNAi (right, 108099KK). **f)** Lifespan of flies expressing poly-$GR_{8}/_{64}$ ± p38α RNAi (52277GD). Error bars denote mean ± SEM (**b-f**).

Statistical analyses: one-way ANOVA and Bonferroni post-hoc multiple comparisons (**b**), two-way ANOVA followed by Tukey's post-hoc multiple comparisons (**c-d**), Kaplan Meyer analysis with log rank (Mantel Cox) test (**e-f**). * = p< 0.05, ***/### = p < 0.001, ****/#### = p < 0.0001. # denote post-hoc comparisons within each time point (**c-d**).

N = (flies) PR_{8} = 3, GR_{8} = 4, GA_{8} = 4, PR_{64} = 5, GR_{64} = 3, GA_{64} = 1 (**b**); 18-20 per genotype, 2 experimental replicates (**c, d**); p38 MAPK 34238GD: PR_{8} = 38, PR_{8} + p38 RNAi = 18, PR_{64} = 39, PR_{64} + p38 RNAi = 31; p38 MAPK 102484KK: PR_{8} = 80, PR_{8} + p38 RNAi = 69, PR_{64} = 80, PR_{64} + p38 RNAi = 81; p38MAPK 108099KK: PR_{8} = 79, PR_{8} + p38 RNAi = 73, PR_{64} = 80, PR_{64} + p38 RNAi = 76 (**e**); GR_{8} = 40, GR_{64} = 59, GR_{8} + p38RNAi = 60, GR_{64} + p38RNAi = 58 (**f**).

Raw data and further statistical information provided in the Source Data File and at doi 10.5281/zenodo.19692872.

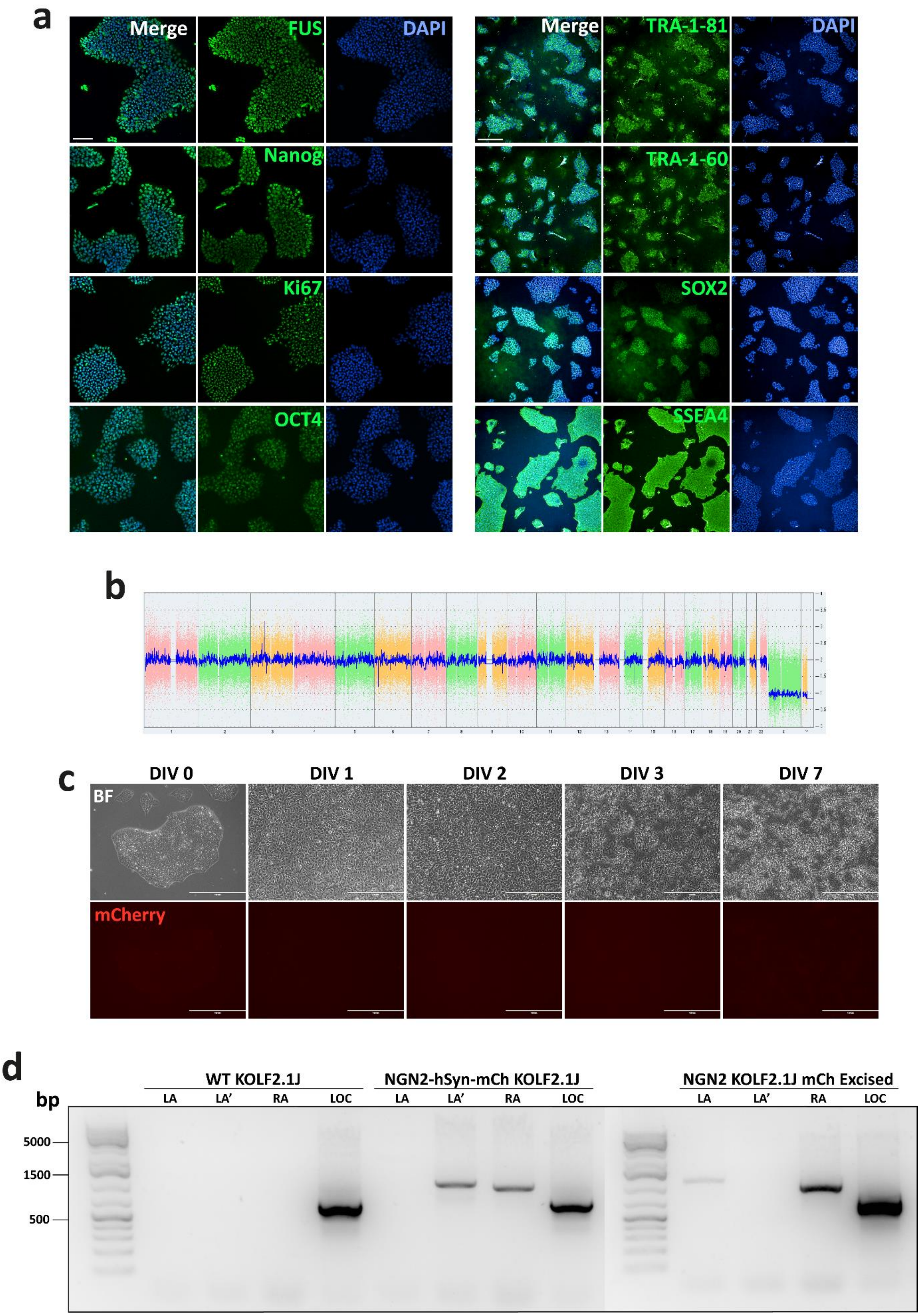
a
Merge
FUS
DAPI
Nanog
Ki67
OCT4
Merge
TRA-1-81
DAPI
TRA-1-60
SOX2
SSEA4
b
c
DIV 0
DIV 1
DIV 2
DIV 3
DIV 7
BF
mCherry
d
bp
5000
1500
500
WT KOLF2.1J
NGN2-hSyn-mCh KOLF2.1J
NGN2 KOLF2.1J mCh Excised
LA
LA'
RA
LOC

Supplementary Figure 9 – KOLF2.1J Wildtype hNGN2 iPSC Line Generation Quality Control

Quality control data for wildtype KOLF2.1J iPSC with a heterozygous doxycycline inducible human neurogenin2 (hNGN2) overexpression cassette inserted at the CYBL locus. **a)** Representative images of pluripotency marker immunofluorescence. **b)** Whole genome view of KaryoStat+ analysis displaying all somatic and sex chromosomes in one frame with a high-level copy number. Pink, green and yellow traces indicate raw signal for each chromosome probe. Blue trace represents normalised probe signal used to identify copy number and aberrations (if any). X axis = chromosome, right Y axis = smoothed log2 ratios of probe signal intensity where 1 = chromosomal loss, 2 = normal copy number state and 3 = chromosomal gain. No aberrations were detected when comparing against the reference data set. **c)** Confirmation of neuronal differentiation capacity and excision of mCherry selection cassette. i3 neuronal differentiation was carried out with doxycyline from DIV0-DIV3, after which point neuronal morphology is clearly visible. mCherry signal is absent, confirming successful excision. **d)** PCR confirming successful CRISPR editing (see methods for primer combinations) and heterozygous allele integrity (‘LOC’).

Scale bars: 100 µm (**a**), 400 µm (**c**).

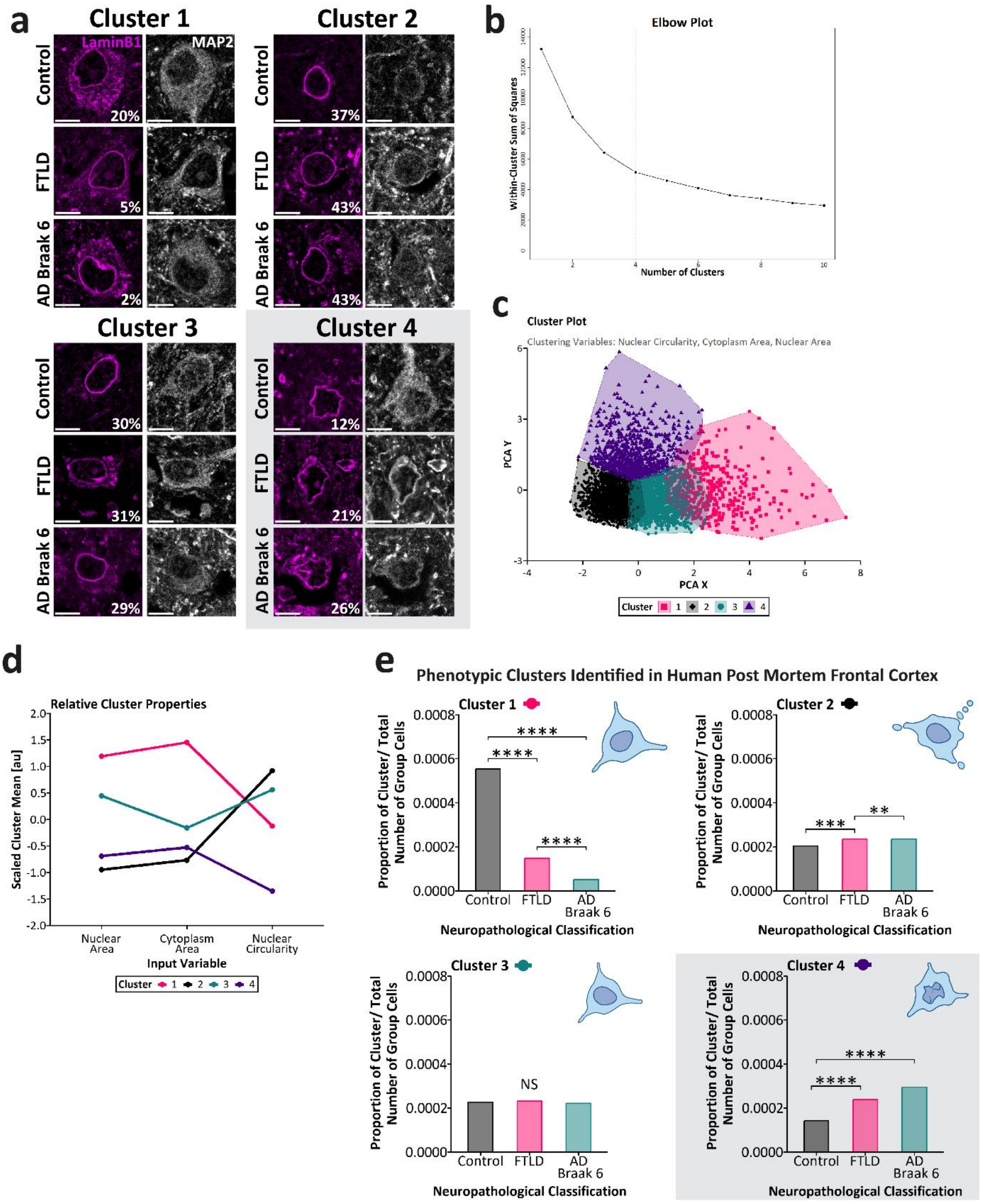

Supplementary Figure 10 – K-means cluster analysis identifies single-cell phenotype clusters from LaminB1 immunofluorescence in post-mortem human frontal cortex

a) Examples of LaminB1 and MAP2 immunofluorescence in single cells from each neuropathological classification group found within each phenotype cluster identified by k means clustering. % = number of cells within the cluster from each group (control, FTLD, AD) shown as a percentage of the total sample for each group (control, FTLD, AD). Scale bar = 10µm. **b**) Elbow plot demonstrating within clusters sum of squares method to model the most appropriate value of *k* to use in k-means cluster analysis for each data set. Vertical dotted line indicates the determined 'elbow' point of the plot, at $k = 4$. **c)** Principal component plot illustrating clusters fitted to data set by k-means cluster analysis. **d)** Parallel coordinates plot visualizing relative differences in mean for each input variable across all clusters identified by k-mean cluster analysis of single neuron LaminB1 immunofluorescence in *post-mortem* brain. 4 input variables: **e)** Frequency bar charts indicating the proportion of neurons from each neuropathological classification group (control, FTLD, AD) within each identified cluster. Proportion is normalised according to overall total number of cells sampled from each neuropathological classification group. Shading highlights the cluster compatible with karyoptotic morphology.

N = 4403 cells, from 27 separate cases (Control = 8, FTLD = 13, AD = 6). Input variables = LaminB1 nuclear circularity, cytoplasm area & nuclear area.

Statistical analyses = χ^2 test followed by pairwise post-hoc binomial test with Bonferronni correction. * = $p < 0.05$, ** = $p < 0.01$, *** = $p < 0.001$, **** = $p < 0.0001$.

Raw data and further statistical information provided in the Source Data File and at doi 10.5281/zenodo.19468523.
