# Supplementary material for: Karyoptosis mediates cell death and neurodegeneration upon proteotoxic stress
Source: Nat Commun. 2026 Jun 25;17:5135. doi: 10.1038/s41467-026-73802-w (PMC13303863; doi:10.1038/s41467-026-73802-w)
Supplement: Supplementary file 2 — Description of Additional Supplementary Files [file 41467_2026_73802_MOESM2_ESM.pdf]

Supplementary Data 1– EV Proteomics

List of all peptides Log2 abundance and relative fold changes and statistical significance for the comparison made. Significantly different peptides in BafA1 vs DMSO control are highlighted in yellow. In green are >2 fold change increases, in red > 2 fold change decreases. In bold are all the proteins annotated as "nucleus" in the UP_KW_CELLULAR_COMPONENT database.

Data is available via ProteomeXchange with identifier PXD074564 at doi 10.6019/PXD074564.

Supplementary Data 2 – LaminB1 peptides

List of all LaminB1 peptides and relative modifications identified by Mass Spectrometry. All phosphopeptides are highlighted in yellow and the peptide containing Ser391 is in bold.

Raw data and further information provided at doi 10.5281/zenodo.19911740

Supplementary Data 3 – Immunofluorescence profiling

Information on human frontal cortex samples and raw data of all cells and their properties for the k-means cluster analyses in human frontal cortex in both datasets, accompanied by quantitative analysis of clusters' weight.
